# ERK1/2 Signaling Dominates Over RhoA Signaling in Regulating Early Changes in RNA Expression Induced by Endothelin-1 in Neonatal Rat Cardiomyocytes

**DOI:** 10.1371/journal.pone.0010027

**Published:** 2010-04-02

**Authors:** Andrew K. Marshall, Oliver P. T. Barrett, Timothy E. Cullingford, Achchuthan Shanmugasundram, Peter H. Sugden, Angela Clerk

**Affiliations:** National Heart and Lung Institute, Faculty of Medicine, Imperial College London, London, United Kingdom; University of Hong Kong, Hong Kong

## Abstract

**Background:**

Cardiomyocyte hypertrophy is associated with changes in gene expression. Extracellular signal-regulated kinases 1/2 (ERK1/2) and RhoA [activated by hypertrophic agonists (e.g. endothelin-1)] regulate gene expression and are implicated in the response, but their relative significance in regulating the cardiomyocyte transcriptome is unknown. Our aim was to establish the significance of ERK1/2 and/or RhoA in the early cardiomyocyte transcriptomic response to endothelin-1.

**Methods/Principal Findings:**

Cardiomyocytes were exposed to endothelin-1 (1 h) with/without PD184352 (to inhibit ERK1/2) or C3 transferase (C3T, to inhibit RhoA). RNA expression was analyzed using microarrays and qPCR. ERK1/2 signaling positively regulated ∼65% of the early gene expression response to ET-1 with a small (∼2%) negative effect, whereas RhoA signaling positively regulated ∼10% of the early gene expression response to ET-1 with a greater (∼14%) negative contribution. Of RNAs non-responsive to endothelin-1, 66 or 448 were regulated by PD184352 or C3T, respectively, indicating that RhoA had a more significant effect on baseline RNA expression. mRNAs upregulated by endothelin-1 encoded a number of receptor ligands (e.g. Ereg, Areg, Hbegf) and transcription factors (e.g. Abra/Srf) that potentially propagate the response.

**Conclusions/Significance:**

ERK1/2 dominates over RhoA in the early transcriptomic response to endothelin-1. RhoA plays a major role in maintaining baseline RNA expression but, with upregulation of Abra/Srf by endothelin-1, RhoA may regulate changes in RNA expression over longer times. Our data identify ERK1/2 as a more significant node than RhoA in regulating the early stages of cardiomyocyte hypertrophy.

## Introduction

Cardiomyocytes are the contractile cells of the heart, constituting ∼70% of the volume and ∼30% of the total cell number. Mammalian cardiomyocytes become terminally-differentiated during the early post-natal period and individual cells grow as the organism grows, a process that may be defined as “eutrophy” [1]. Cardiomyocytes also undergo hypertrophic growth, defined as an increase in cardiomyocyte size above that which occurs at any given stage of post-natal mammalian growth [1], [2]. *In vivo*, cardiomyocyte hypertrophy is an important adaptational response that allows the heart to maintain an adequate cardiac output with improved cardiac contractility in a variety of physiological loading conditions including pregnancy [3] or endurance exercise such as voluntary running [4]. This is probably beneficial and is reversible. In ‘pathological’ conditions (e.g. hypertension or following myocardial infarction), the heart may initially undergo a physiological type of hypertrophy in order to increase or maintain cardiac output. However, this may degenerate possibly because of ancillary changes (e.g. fibrosis, reduced contractile performance and chamber dilatation/wall thinning) leading to “maladaptive” hypertrophy and, potentially, heart failure [5].

Cardiomyocyte hypertrophy is associated with morphological changes (increase in cell size and contractile apparatus) that are facilitated by changes in gene expression and an increase in the rate of protein synthesis [1], [2]. Changes in gene expression include re-expression of genes expressed early in development, the so-called “fetal” programme (e.g. atrial and B-type natriuretic factors, β-myosin heavy chain), expression of immediate early genes (IEGs) such as c-*jun*, c-*fos* and c-*myc*, and changes in expression of proteins associated with contractility [6]. It is assumed that extracellular stimuli activate a programme of intracellular signaling events that leads to the changes in gene expression, increased protein synthesis and the development of a hypertrophic response. Gq protein-coupled receptor (GqPCR) agonists such as endothelin-1 (ET-1) are particularly implicated in promoting hypertrophy [7]. In cardiomyocytes, ET-1 potently activates protein kinase C isoforms, the small G protein Ras and signaling through extracellular signal-regulated kinases 1/2 (ERK1/2) [7]. ET-1 activates other mitogen-activated protein kinases (MAPKs), c-Jun N-terminal kinases (JNKs) and p38-MAPKs, although these are activated more potently by cellular stresses. Rho-family small G proteins (RhoA, Rac1) are also activated by ET-1 and other GqPCR agonists [8]. All these signaling pathways are implicated in cardiomyocyte hypertrophy [6], [8].

Although much is known of the signaling pathways activated by hypertrophic stimuli, less is known of the genes they regulate. MAPKs phosphorylate transcription factors (TFs) to modulate their transactivating activities and regulate IEG expression (e.g. ERK1/2 phosphorylate and activate Elk1 [6]). IEGs encode “structural” proteins that directly influence cell function and “regulatory” proteins. The latter include TFs that regulate downstream gene expression and soluble mediators that act in autocrine/paracrine loops to initiate further cycles of intracellular signaling. RhoA is also particularly implicated in regulating cardiomyocyte gene expression. Stimulation of actin “treadmilling” by RhoA leads to activation of Abra (also known as STARS or MS-1) that promotes nuclear translocation of myocardin-related TFs (MRTFs) [9]. Like Elk1, MRTFs co-operate with serum-response factor (Srf) to promote gene expression [6], [9]. Precisely which genes are regulated by these individual signaling pathways and TFs remains to be established.

We previously mapped the acute, temporal changes in RNA expression induced in cardiomyocytes by ET-1 [10]. A large proportion of the very early changes in IEG expression (30 min) require ERK1/2 or ERK5 signaling (we used U0126 that inhibits the upstream kinases for both pathways [11]), and this is reflected in second-phase gene expression (2–4 h) [10], [12]. However, the majority of the IEG response occurs at ∼1 h [10] and the relative contribution of ERK1/2, RhoA or other signaling pathways is unknown. Here, we used an inhibitor approach to establish the significance of ERK1/2 *vs* RhoA in regulating the baseline and ET-1-responsive cardiomyocyte transcriptome. PD184352 is highly specific for the ERK1/2 cascade when used at 2 µM [11] and C3 transferase (C3T) from *Clostridium botulinum* inactivates only RhoA/B/C family proteins [13]. We conclude that, whereas RhoA signaling is significant in maintaining baseline RNA expression, ERK1/2 signaling is dominant in the acute response to ET-1 and plays a generally positive role in RNA expression.

## Results

**Figure 1:**
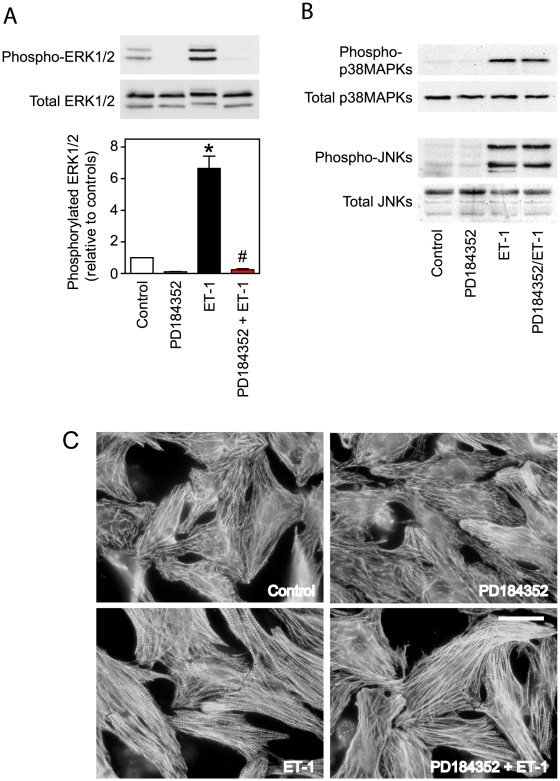
Inhibition of ERK1/2 activation by PD184352. Cardiomyocytes were unstimulated (Control) or exposed to 100 nM ET-1, 2 µM PD184352 or ET-1 in the presence of PD184352. Incubation times with ET-1 were 5 min (for activation of ERK1/2 or p38-MAPK), 15 min (for activation of JNKs) or 1 h (for effects on effects on morphology). A, Phosphorylated (upper panel) or total (center panel) ERK1/2 were assessed by Western blotting. Densitometric analysis is shown in the lower panel as means ± SEM (n = 5 independent experiments). * p<0.001 relative to control, # p<0.001 relative to ET-1 alone (one-way ANOVA with Newman-Keuls post-test). B, Phosphorylated or total p38-MAPKs (upper blots) or JNKs (lower blots) were assessed by Western blotting. The experiment was repeated with similar results. C, Cardiomyocytes were immunostained with antibodies to troponin T. The experiment was repeated with similar results. Bar = 10 µm.

**Figure 2:**
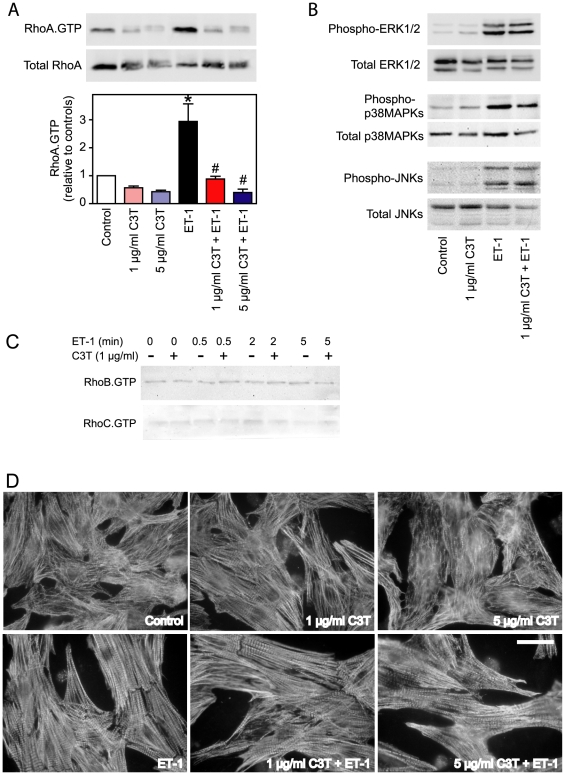
Inhibition of RhoA activation by C3T. Cardiomyocytes were unstimulated (Control) or exposed to 100 nM ET-1, 1 or 5 µg/ml C3T (2 h pretreatment) or ET-1 in the presence of C3T. Incubation times with ET-1 were 30 s (for activation of RhoA), 5 min (for activation of ERK1/2 or p38-MAPK), 15 min (for activation of JNKs) or 1 h (for effects on morphology). A, Affinity purified RhoA.GTP (upper panel) and total RhoA (center panel) were assessed by Western blotting. Blots are representative of 4 independent experiments. Densitometric analysis is provided in the lower panels. Results are means ± SEM (n = 4). * p<0.001 relative to controls, # p<0.001 relative to ET-1 alone (one way ANOVA with Newman-Keuls post-test). B, Phosphorylated or total ERK1/2 (upper blots), p38-MAPKs (center blots) or JNKs (lower blots) were assessed by Western blotting. The experiment was repeated with similar results. C, Cardiomyocytes were exposed to ET-1 for the times indicated with or without pretreatment with C3T. Affinity purified RhoB.GTP (upper image) and RhoC.GTP (lower image) were assessed by Western blotting. The experiment was repeated with similar results. D, Cardiomyocytes were immunostained with antibodies to troponin T. The experiment was repeated with similar results. Bar = 10 µm.

PD184352 (2 µM) inhibited baseline ERK1/2 activity and the increase induced by ET-1 (Fig. 1A). There was no effect of PD184352 on baseline activities of p38-MAPKs or JNKs, or on the increase induced by ET-1 (Fig. 1B). PD184352 alone had no overt effect on cardiomyocyte morphology as determined by immunostaining for troponin T (i.e. myofibrillar structure), but caused some reduction in the increase in myofibrillar organisation induced by ET-1 (Fig. 1C). C3T (1 µg/ml) inhibited baseline RhoA.GTP and the activation of RhoA by ET-1 (Fig. 2A). At 5 µg/ml, C3T promoted a further reduction in RhoA.GTP and reduced the rate of migration through polyacrylamide gels. C3T (1 µg/ml) had no effect on basal MAPK activities or on activation of MAPKs by ET-1 (Fig. 2B). Although we detected RhoB.GTP and RhoC.GTP in cardiomyocyte extracts, we did not detect any increase in response to ET-1 and C3T had no effect on basal levels in the affinity purification assay (Fig. 2C). The effects of C3T on cardiomyocyte RNA expression may therefore be presumed to be mediated through RhoA. Although RhoA modulates actin structures, 1 µg/ml C3T had no overt effects on cardiomyocyte myofibrillar structure or on the increase in myofibrillar organisation induced by ET-1 (Fig. 2D). However, 5 µg/ml C3T caused some reduction in myofibrillar content with disruption of myofibrillar structure both in the basal state and following treatment with ET-1. 1 µg/ml C3T was used for all further experiments given that this was sufficient to inhibit RhoA.GTP in the absence of further modification of the protein (as indicated by reduced gel mobility) or disruption of cardiomyocyte structure.

**Figure 3:**
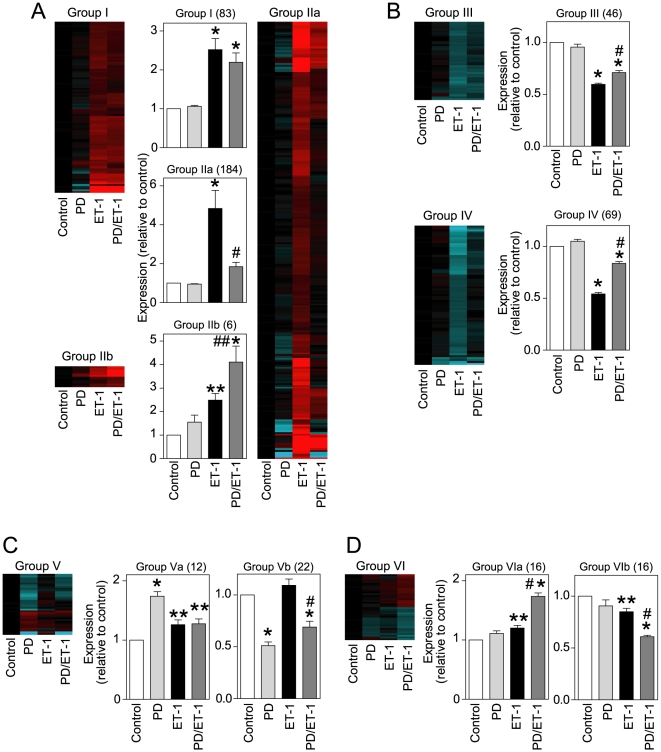
Regulation of cardiomyocyte RNA expression through the ERK1/2 cascade. Cardiomyocytes were unstimulated (Control) or exposed to ET-1, PD184352 (PD) or ET-1 together with PD184352 (PD/ET-1). Changes in RNA expression were determined using microarrays using GeneSpring analysis to identify transcripts with significant changes in expression (>1.5-fold) with or without a significant effect of the inhibitor (one-way ANOVA with Newman-Keuls post-test and Benjamini-Hochberg false discovery rate correction). A, Upregulated by ET-1 with no effect (Group I), inhibition (Group IIa) or enhancement by PD184352 (Group IIb). B, Downregulated by ET-1 with no effect (Group III) or inhibition by PD184352 (Group IV). C, Upregulation (Group Va) or downregulation (Group Vb) by PD184352. D, Upregulation (Group VIa) or downregulation (Group VIb) by PD/ET-1. Heatmaps represent all probesets in each group [Log2 scale; -2.5 (cyan) through 0 (black) to 2.5 (red)]. Histograms are means ± SEM (numbers of transcripts in parentheses). Statistical analysis of each group was performed using GraphPad Prism 4: * p<0.001, **p<0.05 relative to Control; # p<0.001, relative to ET-1 alone (one-way ANOVA with Newman-Keuls post-test).

**Figure 4:**
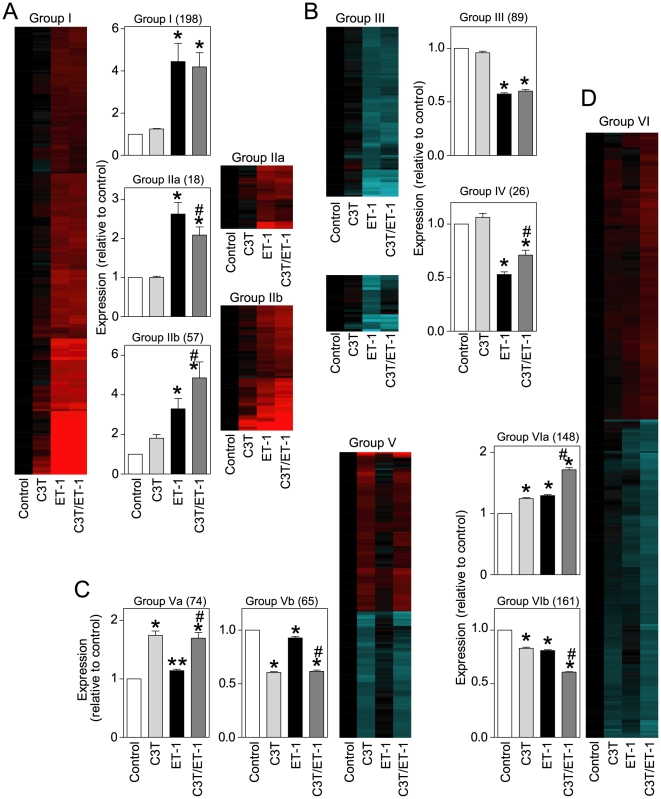
Regulation of cardiomyocyte RNA expression through RhoA. Cardiomyocytes were unstimulated (Control) or exposed to ET-1, C3T or ET-1 together with C3T (C3T/ET-1). Changes in RNA expression were determined using microarrays using GeneSpring analysis to identify transcripts with significant changes in expression (>1.5-fold) with or without a significant effect of the inhibitor (one-way ANOVA with Newman-Keuls post-test and Benjamini-Hochberg false discovery rate correction). A, Upregulated by ET-1 with no effect (Group I), inhibition (Group IIa) or enhancement by C3T (Group IIb). B, Downregulated by ET-1 with no effect (Group III) or inhibition by C3T (Group IV). C, Upregulation (Group Va) or downregulation (Group Vb) by C3T. D, Upregulation (Group VIa) or downregulation (Group VIb) by C3T/ET-1. Heatmaps are shown for probesets in each group [Log2 scale; -2.5 (cyan) through 0 (black) to 2.5 (red)]. Histograms are means ± SEM (numbers of transcripts in parentheses). Statistical analysis of each group was performed using GraphPad 4: * p<0.001, **p<0.05 relative to Control; # p<0.001, relative to ET-1 alone (one-way ANOVA with Newman-Keuls post-test).

To determine the significance of ERK1/2 signaling *vs* RhoA signaling in regulating cardiomyocyte RNA expression, cardiomyocytes were exposed to C3T, PD184352 or ET-1 (1 h) alone, or C3T or PD184352 with ET-1. Transcriptional profiling was performed with Affymetrix Rat Genome 230 2.0 microarrays using GeneSpring for analysis. Of 16285 probesets detecting RNAs in cardiomyocytes, 1323 were significantly changed in any condition (PD184352, C3T, ET-1, PD184352/ET-1, C3T/ET-1) relative to controls (>1.5-fold change; false discovery rate, FDR<0.05). These were clustered according to ET-1 response and the effects of PD184352 (Fig. 3) or C3T (Fig. 4).

Of 273 RNAs upregulated by ET-1, 83 were unaffected by PD184352, whereas upregulation of 184 or 6 was either inhibited or enhanced, respectively (Fig. 3A, Table S1). Of 115 RNAs downregulated by ET-1, the downregulation of 69 was significantly inhibited by PD184352 (Fig. 3B, Table S2). PD184352 had minor effects on baseline RNA expression with few significantly upregulated or downregulated by PD184352 alone or (despite a negligible effect of ET-1 alone) together with ET-1 (28 upregulated; 38 downregulated) (Fig. 3, C and D; Table S3). Thus, ERK1/2 signaling positively regulates ∼65% of the IEG response to ET-1 with a small (∼2%) negative effect, and plays a minor role in regulating baseline gene expression. For C3T, the patterns were markedly different. Of the RNAs upregulated by ET-1, most were unaffected by C3T (198), 18 were significantly inhibited to a minor degree (20.1% average inhibition) and the upregulation of 57 RNAs was significantly enhanced (Fig. 4A, Table S4). Of RNAs downregulated by ET-1, the response for 26 was significantly inhibited (Fig. 4B, Table S5). C3T profoundly affected baseline RNA expression alone (74 upregulated; 65 downregulated) or, despite a negligible effect of ET-1 alone, together with ET-1 (148 upregulated; 161 downregulated) (Fig. 4, C and D, Table S6). Thus, RhoA signaling positively regulates ∼10% of the IEG response to ET-1 with negative regulation of ∼14% of the response. However, it appears to have a significant role in regulating baseline gene expression.

**Figure 5:**
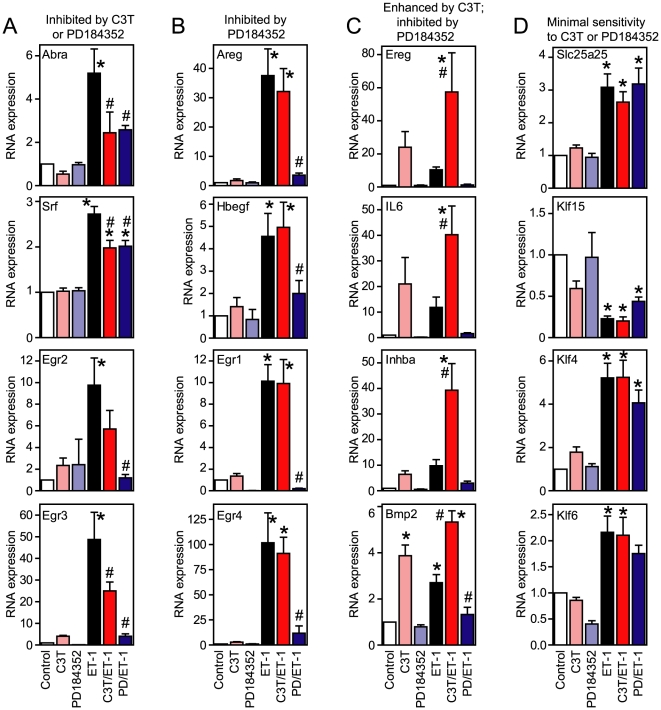
Validation of microarray data. Cardiomyocytes were unstimulated (Control) or exposed to ET-1, C3T, PD184352 or ET-1 together with C3T (C3T/ET-1) or PD184352 (PD/ET-1). ET-1 responsive mRNA expression was measured by qPCR. A, mRNAs inhibited by either C3T or PD184352. B, mRNAs inhibited by PD184352. C, mRNAs enhanced by C3T but inhibited by PD184352. D, mRNAs insensitive to either drug. Results (relative to controls) are means ± SEM (n = 4 or 5). *p<0.05 relative to Control; #p<0.05 relative to ET-1 (one way ANOVA repeated measures with Newman-Keuls post-test).

**Figure 6:**
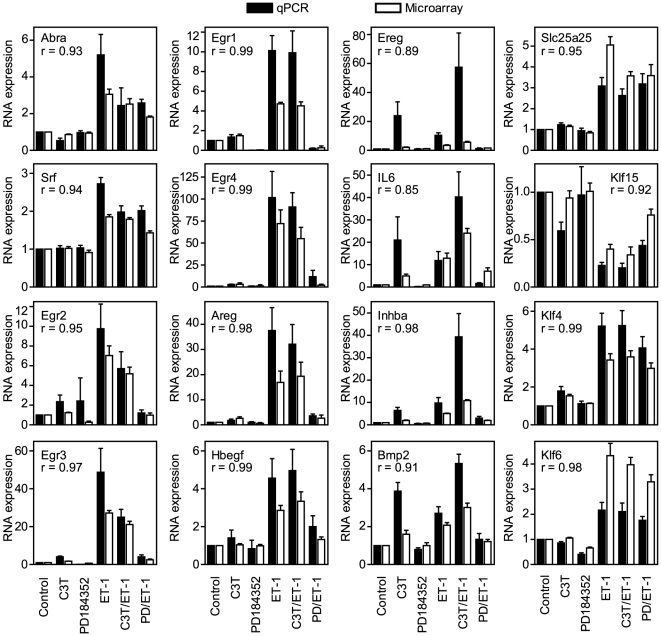
Comparison of qPCR and microarray data. Cardiomyocytes were unstimulated (Control) or exposed to ET-1, C3T, PD184352 or ET-1 together with C3T (C3T/ET-1) or PD184352 (PD/ET-1). ET-1 responsive mRNA expression measured by qPCR (see Figure 5 for detailed representations) was compared with the microarray data (solid bars = qPCR; open bars = microarray data). Results are relative to controls and are means ± SEM (n = 4 or 5). The linear regression coefficient is provided (r) for qPCR *vs* microarray data.

**Figure 7:**
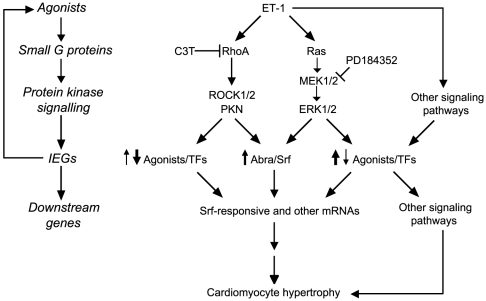
Schematic of signaling and gene expression responses to ET-1 in cardiomyocytes. Agonists (e.g. ET-1) activate intracellular signaling pathways including small G proteins and protein kinases. C3T and PD184352 inhibit activation of RhoA and ERK1/2, respectively. Intracellular signaling pathways modulate expression of immediate early genes (IEGs) including Abra/Srf that participate in RhoA-mediated regulation of cardiomyocyte hypertrophy. These and other transcription factors (TFs) promote expression of downstream genes. Agonists are also produced that act in autocrine/paracrine loops to initiate further cycles of signaling/gene expression. These networks eventually culminate in hypertrophy.

The microarray data were validated by qPCR. We selected ET-1 responsive mRNAs that were inhibited by either PD184352 or C3T (Fig. 5A), inhibited by PD184352 alone (Fig. 5B), enhanced by C3T but inhibited by PD184352 (Fig. 5C) or with minimal sensitivity to either inhibitor (Fig. 5D). We focused mainly on mRNAs encoding TFs (Abra, Srf, Egr1, Egr2, Egr3, Egr4, Klf15, Klf4 and Klf6) and receptor ligands (Areg, Bmp2, Ereg, Hbegf, IL6, Inhba), including Slc25a25 as an additional transcript with low sensitivity to PD184352 or C3T. Absolute relative changes differ with the two techniques [10] but, qualitatively, the qPCR data showed a high degree of correlation with the microarray results (Fig. 6). The data illustrate potential autocrine/paracrine and transcriptional networks operating downstream from the initial stimulus, ET-1 (Fig. 7).

## Discussion

Cardiac hypertrophy is a multifaceted, complex disorder where various stimuli and signals conspire to initiate and propagate disease development. This is presumably reflected at the cardiomyocyte level, and a catalog of potential components of the hypertrophic response has developed [6]. Confusingly, almost all stimuli and signaling pathways are implicated in various aspects of hypertrophy. The challenge is to integrate this information and identify the key nodes. Here, we used highly selective inhibitors (PD184352 and C3T) to establish the global roles of ERK1/2 *vs* RhoA in regulating cardiomyocyte RNA expression. We identified ERK1/2 as a significant node in the IEG response to ET-1, particularly with respect to positive regulation of RNA expression, whereas RhoA has a less significant role with positive and negative effects (Figs. 3 and 4). As might be expected, some RNAs were not significantly regulated by either ERK1/2 or RhoA (Fig. 5D), implicating additional pathways in the cardiomyocyte IEG response to ET-1. These may include other MAPKs (JNKs and p38-MAPKs) that are activated by ET-1 [7]. p38-MAPK is implicated in regulating mRNA stability through Zfp36 [14], a protein which is expressed in cardiomyocytes and upregulated by ET-1 [10], and further studies are required to assess the importance of a p38-MAPK-Zfp36 signal in our system. Our conclusions are summarized in Fig. 7.

Our data with PD184352 are consistent with other studies also identifying ERK1/2 as a key node in the early response to growth stimuli, platelet-derived growth factor and Egf [15], [16]. However, other studies have suggested that RhoA plays a more positive role in regulating RNA expression of, for example Klf2 [17] CTGF [18], Bax [19] or Cyr61 [20]. Although Klf2, CTGF, and Cyr61 (but not Bax) were upregulated by ET-1, this was not inhibited by C3T and C3T alone did not influence Bax expression (Fig. 4, Tables S4 and S6). The reasons may reflect the methods used, times studied, stimulus and/or specific cell types. Some studies emphasise the use of hydroxymethylglutaryl CoA reductase inhibitors (“statins”) as inhibitors of Rho-family signaling but these inhibitors have a range of effects apart from inhibition of RhoA signaling [21]. Notably, we selected the minimum time and concentration of a cell-permeable form of C3T to inhibit RhoA. Other studies used higher concentrations with prolonged incubation that may have secondary effects. Interestingly, ERK1/2 and RhoA converged to increase expression of Abra and Srf mRNAs (Fig. 5A). RhoA signaling may therefore be of greater significance in positively regulating RNA expression over longer times.

One dimension that can confer additional structure to the system is time, and the timing/duration of signaling and gene expression responses is likely to influence the cellular response. Thus, a single stimulus (e.g. ET-1) may initiate hypertrophy but, since signaling and gene expression responses are rapid and transient, other factors, acting at later times may be required for a fully developed response. In addition to influencing TF expression, ET-1 and other stimuli modulate expression of a range of paracrine/autocrine factors including Egf receptor ligands (e.g. amphiregulin, Areg; epiregulin, Ereg, HbEGF [22]), gp130 ligands (e.g. interleukin 6, IL6; leukemia inhibitory factor, Lif [23]) and ligands of the transforming growth factor (Tgf) β family (e.g. inhibin βA, Inhba; bone morphogenetic protein 2, Bmp2; Tgfb3 [24]) (Fig. 5, Table S1) each of which triggers specific receptor subtypes to activate specific intracellular signaling pathways. These, together with major changes in TF expression (e.g. Srf, Egr family, Klf family TFs; Fig. 5) are likely to propagate the response. With a likely scenario *in vivo* being low-level exposure to various stimuli, we suggest that such networks facilitate progression from a multiplicity of initiation points to a similar end-stage hypertrophic phenotype. Understanding these networks and the links between stimuli, signaling, TFs and gene expression is a systems biology challenge for the future.

## Materials and Methods

### Ethics statement

Sprague-Dawley female rats with 2–4 day litters were purchased from Harlan SeraLab Ltd. UK and were housed overnight in the Imperial College Central Biomedical Services facility with water and food *ad libitum*. Both facilities are UK registered with Home Office certificates of designation. All procedures in these facilities were performed in accordance with UK regulations and the European Community Directive 86/609/EEC for animal experiments. Animals were culled by schedule 1 (cervical dislocation) for which additional approval and licences are not required according to UK regulations.

### Cardiomyocyte cultures

Ventricles were dissected from neonatal (1–2 d) Sprague-Dawley rat hearts and dissociated by serial digestion with 0.4 mg/ml collagenase and 0.6 mg/ml pancreatin sterile digestion buffer (116 mM NaCl, 20 mM HEPES, 0.8 mM Na_2_HPO_4_, 5.6 mM glucose, 5.4 mM KCl and 0.8 mM MgSO_4_, pH 7.35). The first digestion supernatant (5 min, 37°C, 160 cycles/min in a shaking waterbath) was removed and discarded. Cell suspensions from subsequent digestions (20 min, 2×25 min, 20 min, 10 min; 37°C 136 cycles/min shaking) were recovered by centrifugation (5 min, 60×g) and the cell pellet resuspended in plating medium (Dulbecco's modified Eagle's medium (DMEM)/medium 199 [4:1 (v/v)], 15% (v/v) FCS, 100 units/ml penicillin and streptomycin). The cells were pre-plated on plastic tissue culture dishes (30 min) to remove non-cardiomyocytes. For biochemistry and molecular biology experiments, non-adherent viable cardiomyocytes were plated at a density of 4×10^6^ cells/dish on 60 mm Primaria dishes pre-coated with sterile 1% (w/v) gelatin (Sigma-Aldrich UK). After 18 h myocytes were confluent and beating spontaneously. For immunostaining experiments, cardiomyocytes were plated at 1.5×10^6^ cells/dish on 35 mm Primaria dishes containing glass coverslips pre-coated with sterile 1% (w/v) gelatin followed by laminin (20 µg/ml in PBS; Sigma-Aldrich UK). The plating medium was withdrawn and cells were incubated in serum-free maintenance medium (DMEM/medium [4:1 (v/v)], 100 units/ml penicillin and streptomycin) for a further 24 h.

Cardiomyocytes were unstimulated (Controls), exposed to PD184352 (2 µM), a cell-permeable form of the exoenzyme C3 transferase from *Clostridium botulinum* (C3T; 1 or 5 µg/ml) or ET-1 (100 nM), or exposed to ET-1 following pretreatment with PD184352 (10 min) or C3T (120 min). PD184352 (Alexis Biochemicals, Enzo Life Sciences) was prepared as a stock solution in DMSO (4 mM). Cell-permeable C3T (Cytoskeleton Inc., Cat. no. CT03) was resuspended in 66% (v/v) glycerol (0.02 mg/ml). This represents highly purified C3T linked to a cell penetrating moiety though a disulfide bond that allows rapid uptake into cells. In the cytoplasm, the disulfide bond is reduced and the C3T diffuses through the cell. ET-1 (Bachem UK) was prepared as a stock solution in water (0.1 mM). ET-1, PD184352 or C3T were added directly to the tissue culture medium. Unless otherwise stated, incubation times with ET-1 were dependent upon the time at which individual signaling components are substantially activated: 30 s for RhoA.GTP loading [25], 5 min for activation of ERK1/2 [26] or p38-MAPKs [27], 15 min for activation of JNKs [28] and 1 h for expression of immediate early genes [10]. For inhibitor studies, cells were pre-treated with inhibitor for a minimum period required to provide adequate inhibition of the pathways (10 min for PD184352; 2 h for C3T) prior to addition of ET-1 for the appropriate times. Cells were also exposed to inhibitor alone for the full duration of the incubation time. All samples were harvested together at the end of the experiment.

### Assays for activated RhoA/B/C.GTP and phosphorylated MAPKs

For the study of RhoA/B/C GTP-loading, a GST-fusion protein was prepared containing residues 7–89 of murine rhotekin (GST-RBD) [25]. Cardiomyocytes were washed with ice-cold PBS and scraped into Buffer A [20 mM Tris-HCl (pH 7.4), 2 mM EDTA, 100 mM KCl, 5 mM MgCl_2_, 5 mM NaF, 0.2 mM Na_3_VO_4_, 0.002 mM microcystin, 10% (v/v) glycerol, 1% (v/v) Triton X-100, 0.5% (v/v) 2-mercaptoethanol, 10 mM benzamidine, 0.2 mM leupeptin, 0.01 mM trans-epoxy succinyl-l-leucylamido-(4-guanidino)butane, 0.3 mM phenylmethylsulfonyl fluoride]. Lysates were centrifuged (10,000×g, 5 min, 4°C). A sample of the supernatant was taken to assess total levels of RhoA/B/C (input). Remaining extracts were incubated with mixing (4°C, 24 h) with GST-RBD that had been previously bound to glutathione-Sepharose beads resuspended in Buffer A. Beads were washed with Buffer A and boiled with SDS-polyacrylamide gel electrophoresis sample buffer (0.33 M Tris-HCl pH 6.8, 10% (w/v) SDS, 13% (v/v) glycerol, 133 mM dithiothreitol, 0.2 mg/mL bromphenol blue). The input and eluted proteins were analyzed by Western blotting using 12% (w/v) polyacrylamide gels.

For analysis of phosphorylated MAPKs, cardiomyocytes were washed with ice-cold PBS and scraped into 150 µl buffer B [20 mM β-glycerophosphate (pH 7.5), 50 mM NaF, 2 mM EDTA, 0.004 mM microcystin LR, 1% (v/v) Triton X-100, 5 mM dithiothreitol, 10 mM benzamidine, 0.2 mM leupeptin, 0.01 mM trans-epoxy succinyl-l-leucylamido-(4-guanidino)butane, 0.3 mM phenylmethylsulfonyl fluoride]. Extracts were centrifuged (5 min, 10,000×g, 4°C), the supernatants were removed, and 150 µl was boiled with 50 µl sample buffer. Total and phosphorylated MAPKs were analyzed by Western blotting using 10% (w/v) polyacrylamide gels.

### Western blotting

Proteins were separated by SDS-polyacrylamide gel electrophoresis on 10% or 12% (w/v) polyacrylamide gels and transferred electrophoretically to nitrocellulose. Nonspecific binding sites were blocked with 5% (w/v) nonfat milk powder in 20 mM Tris-HCl pH 7.5, 137 mM NaCl, 0.1% (v/v) Tween 20 (TBST) for 30 min. Blots were incubated with primary antibodies (1∶1000 dilution in TBST containing 5% (w/v) bovine serum albumin, overnight, 4°C). Mouse monoclonal antibodies to RhoA (sc-418), RhoB (sc-8048) and RhoC (sc-12116) were from Santa Cruz Biotechnology Inc. Rabbit antibodies to dually-phosphorylated ERK1/2 (Cat. no. 4377), total ERK1/2 (Cat. no. 9102), dually-phosphorylated p38-MAPKs (Cat. no. 9216), total p38-MAPKs (Cat. no. 9212), dually-phosphorylated JNKs (Cat. no. 4671) and total JNKs (Cat. no. 9252) were from Cell Signaling. All antibodies were used at 1/1000 dilution. The blots were washed with TBST (three times for 5 min, room temperature), incubated with polyclonal secondary antibodies conjugated to horseradish peroxidase (Dako; 1∶5000 dilution in TBST containing 1% (w/v) nonfat milk powder, 1 h, room temperature) and then washed again in TBST (three times for 5 min, room temperature). Bands were detected by enhanced chemiluminescence using ECL Plus Western Blotting detection reagents (GE Healthcare) with visualisation using an ImageQuant 350 digital imager (GE Healthcare). ImageQuant 7.0 software (GE Healthcare) was used for densitometric analysis of the bands.

### Immunostaining

Cells were washed with ice-cold PBS and fixed in 3.7% (v/v) formaldehyde in PBS (10 min, room temperature). Cardiomyocytes were permeabilised with 0.1% (v/v) Triton X-100 (10 min, room temperature) in PBS and non-specific binding blocked with 1% (w/v) bovine serum albumin in PBS containing 0.1% (v/v) Triton X-100 (10 min, room temperature). All incubations were at 37°C in a humidified chamber, and coverslips were washed three times in PBS after each stage of the immunostaining procedure. Cardiomyocytes were stained with mouse monoclonal primary antibodies to troponin T (1/40, 60 min; Stratech Scientific, Cat. no. MS-295-P1) with anti-mouse immunoglobulin secondary antibodies coupled to Alexa-Fluor 488 (1/200, 60 min; Invitrogen). Coverslips were mounted using fluorescence mounting medium (Dako) and viewed with a Zeiss Axioskop fluorescence microscope using a 100× oil-immersion objective. Digital images captured using a Canon PowerShot G3 camera were converted to grayscale and reduced in size using Adobe Photoshop 7.0.

### RNA preparation and microarray analysis

Cardiomyocytes were unstimulated (Controls, 2 per preparation), exposed to ET-1 (1 h), PD184352 (70 min) or C3T (3 h), or exposed to ET-1 (1 h) following pretreatment with PD184352 (10 min) or C3T (2 h). Total RNA was extracted using RNA Bee (1 ml per 4×10^6^ cells, AMS Biotechnology Ltd) according to the manufacturer's instructions. The purity was assessed from the A_260_/A_280_ (values of 1.9–2.1 were considered acceptable). RNA concentrations were determined from the A_260_. To minimise variation resulting from different cardiomyocyte preparations, equal amounts of RNA from three individual experiments were pooled to generate a single sample set. First-strand cDNA synthesis was performed using 10 µg total RNA with a T7-(dT)_24_ primer and Superscript II (Invitrogen) (37°C, 1 h). Second-strand cDNA synthesis was carried out using E. coli DNA ligase, E. coli DNA polymerase I, and RNase H (Invitrogen) (2 h, 16°C). Biotin-labeled antisense cRNA was synthesized from purified double-stranded cDNA using the BioArray High Yield RNA transcript labelling kit (Enzo Diagnostics) according to the manufacturer's instructions. Four sets of pooled samples were prepared for hybridization to separate Affymetrix rat genome 230 2.0 arrays (i.e. four separate sets of samples were analysed for each condition, prepared from a total of 12 myocyte preparations). Two separate unstimulated controls were prepared and hybridized simultaneously with each set of samples. Fragmentation of antisense cRNA and hybridization to Affymetrix rat genome 230 2.0 arrays was performed at the CSC/IC Microarray Centre according to the manufacturer's instructions. MIAME-compliant data were exported to ArrayExpress (ArrayExpress ID: E-MEXP-1977 and E-MEXP-2516).

Data (.CEL files) were imported into GeneSpring 10.0.2 (Agilent Technologies) and were normalized using the MAS5 algorithm. Normalisation per gene was to the two corresponding controls within each sample set. Probesets were selected for analysis if present or marginal in all controls or all of any of the treatments, and filtered according to fold change (>1.5-fold) with any condition (ET-1, C3T, PD184352, C3T/ET-1 or PD184352/ET-1) relative to controls. Probesets with statistically significant changes (false discovery rate, FDR<0.05) were identified by one-way ANOVA with Student-Newman-Keuls (SNK) post test, applying a Benjamini and Hochberg multiple testing correction. Supervised clustering was performed. RNAs were clustered according to >1.5-fold change with ET-1 (Group A), >1.5-fold change with PD184352 or PD184352/ET-1 (Group B), >1.5-fold change with C3T or C3T/ET-1 (Group C). Group A was clustered according to upregulation or downregulation with ET-1 and significant difference (inhibition or enhancement) for ET-1 *vs* PD184352/ET-1 or C3T/ET-1. Groups B and C were clustered according to upregulation or downregulation with inhibitor alone or inhibitor plus ET-1. Gene identities for all selected probesets were confirmed by BLAST search of probeset sequences using the Entrez nucleotide database (www.ncbi.nlm.nih.gov/BLAST). Further BLAST searches for unassigned sequences were performed against the rat genome (www.ncbi.nlm.nih.gov/genome/seq/BlastGen/BlastGen.cgi?taxid=10116) and the mouse genome (www.ncbi.nlm.nih.gov/genome/seq/BlastGen/BlastGen.cgi?taxid=10090; cross-species megaBLAST) (since the rat genome is less well annotated). Genes were classified as far as possible using GeneOntology classifications associated with rat, mouse and human orthologs (NCBI Entrez Gene; www.ncbi.nlm.nih.gov/entrez), taking into account both probable Function and Process. For genes with conflicting potential functions, further searches were performed using PubMed (www.ncbi.nlm.nih.gov/sites/entrez?db=pubmed) to ascertain probable biochemical function.

### Validation of microarray results by qPCR

Cardiomyocytes were treated and total RNA extracted as for microarray analysis. cDNAs were synthesized using High Capacity cDNA Reverse Transcription Kits with random primers (Applied Biosystems) according to the manufacturer's instructions. For selected genes, gene sequences were obtained from NCBI Entrez Gene and information on gene structures was obtained from Ensembl (www.emsembl.org). Primers were designed for qPCR with an amplicon size of 50-150 bp, within 1000 bp of the 3′ end of the mRNA and, where possible, across an exon boundary (Table S7). qPCR was performed using an ABI Real-Time PCR 7500 system (Applied Biosystems). Optical 96-well reaction plates were used containing (in each well) 12.5 µl SYBR Green Jump Start Taq Readymix (Sigma-Aldrich UK), 5 µl oligonucleotide primers (5 pmol each of forward and reverse primers) and 7.5 µl (1 µg) cDNA template. qPCR was performed using absolute quantification with the standard curve protocol. Dissociation curve analysis was routinely performed to identify any aberrant amplification products. Values for selected RNAs were normalized to glyceraldehyde 3-phosphate dehydrogenase (Gapdh) expression and then to control values.

## Supporting Information

Table S1 RNAs upregulated in cardiomyocytes by ET-1: effects of PD184352. Cardiomyocytes were unstimulated (Control) or exposed to ET-1, PD184352 (PD) or ET-1 in the presence of PD184352 (PD/ET-1). Microarray analysis was performed to identify RNAs significantly upregulated by ET-1 (>1.5-fold change, FDR<0.05) and with significant inhibition or enhancement with PD184352. Raw values are provided for Controls and expression relative to Controls is provided for PD, ET-1 and PD/ET-1. Results are means for 4 separate hybridisations. Where multiple probesets represented the same RNA, individual raw values are provided for controls and, since the relative fold changes were similar, the mean values are provided for the treatments. RNAs in each group (inhibited by PD184352, enhanced by PD184352, no significant effect of PD184352) are listed alphabetically according to gene symbol.
(0.51 MB DOC)

Table S2 RNAs downregulated in cardiomyocytes by ET-1: effects of PD184352. Cardiomyocytes were unstimulated (Control) or exposed to ET-1, PD184352 (PD) or ET-1 in the presence of PD184352 (PD/ET-1). Microarray analysis was performed to identify RNAs significantly downregulated by ET-1 (>1.5-fold change, FDR<0.05) and with significant inhibition or enhancement with PD184352. Raw values are provided for Controls and expression relative to Controls is provided for PD, ET-1 and PD/ET-1. Results are means for 4 separate hybridisations. Where multiple probesets represented the same RNA, individual raw values are provided for controls and, since the relative fold changes were similar, the mean values are provided for the treatments. RNAs in each group (inhibited by PD184352, no significant effect of PD184352) are listed alphabetically according to gene symbol.
(0.23 MB DOC)

Table S3 RNAs regulated in cardiomyocytes by PD184352 alone or PD184352 in the presence of ET-1. Cardiomyocytes were unstimulated (Control) or exposed to ET-1, PD184352 (PD) or ET-1 in the presence of PD184352 (PD/ET-1). Microarray analysis was performed. RNAs not significantly regulated by ET-1 alone but significantly regulated by PD or PD/ET-1 were selected (>1.5-fold change, FDR<0.05) and clustered according to upregulation or downregulation. Raw values are provided for Controls and expression relative to Controls is provided for PD, ET-1 and PD/ET-1. Results are means for 4 separate hybridisations. Where multiple probesets represented the same RNA, individual raw values are provided for controls and, since the relative fold changes were similar, the mean values are provided for the treatments. RNAs in each group are listed alphabetically according to gene symbol.
(0.15 MB DOC)

Table S4 RNAs upregulated in cardiomyocytes by ET-1: effects of C3T. Cardiomyocytes were unstimulated (Control) or exposed to ET-1, C3T or ET-1 in the presence of C3T (C3T/ET-1). Microarray analysis was performed to identify RNAs significantly upregulated by ET-1 (>1.5-fold change, FDR<0.05) and with significant inhibition or enhancement with C3T. Raw values are provided for Controls and expression relative to Controls is provided for C3T, ET-1 and C3T/ET-1. Results are means for 4 separate hybridisations. Where multiple probesets represented the same RNA, individual raw values are provided for controls and, since the relative fold changes were similar, the mean values are provided for the treatments. RNAs in each group (inhibited by C3T, enhanced by C3T, no significant effect of C3T) are listed alphabetically according to gene symbol.
(0.50 MB DOC)

Table S5 RNAs upregulated in cardiomyocytes by ET-1: effects of C3T. Cardiomyocytes were unstimulated (Control) or exposed to ET-1, C3T or ET-1 in the presence of C3T (C3T/ET-1). Microarray analysis was performed to identify RNAs significantly downregulated by ET-1 (>1.5-fold change, FDR<0.05) and with significant inhibition or enhancement with C3T. Raw values are provided for Controls and expression relative to Controls is provided for C3T, ET-1 and C3T/ET-1. Results are means for 4 separate hybridisations. Where multiple probesets represented the same RNA, individual raw values are provided for controls and, since the relative fold changes were similar, the mean values are provided for the treatments. RNAs in each group (inhibited by C3T, no significant effect of C3T) are listed alphabetically according to gene symbol.
(0.23 MB DOC)

Table S6 RNAs regulated in cardiomyocytes by C3T alone or C3T in the presence of ET-1. Cardiomyocytes were unstimulated (Control) or exposed to ET-1, C3T or ET-1 in the presence of C3T (C3T/ET-1). Microarray analysis was performed. RNAs not significantly regulated by ET-1 alone but significantly regulated by C3T or C3T/ET-1 were selected (>1.5-fold change, FDR<0.05) and clustered according to upregulation or downregulation. Raw values are provided for Controls and expression relative to Controls is provided for C3T, ET-1 and C3T/ET-1. Results are means for 4 separate hybridisations. Where multiple probesets represented the same RNA, individual raw values are provided for controls and, since the relative fold changes were similar, the mean values are provided for the treatments. RNAs in each group are listed alphabetically according to gene symbol.
(0.84 MB DOC)

Table S7 Primers used for qPCR validation of microarray data. Nucleotide positions in transcripts are shown in parentheses for each primer. mRNA sequences (accession numbers provided) for established genes were obtained from the Rat Genome Database (http://rgb.mcw.edu, viewed at http://www.ncbi.nlm.nih.gov/entrez).
(0.01 MB PDF)
