# Supplementary material for: ERK1/2 Signaling Dominates Over RhoA Signaling in Regulating Early Changes in RNA Expression Induced by Endothelin-1 in Neonatal Rat Cardiomyocytes
Source: PLoS One. 2010 Apr 2;5(4):e10027. doi: 10.1371/journal.pone.0010027 (PMC2848868; doi:10.1371/journal.pone.0010027)
Supplement: Table S1 — RNAs upregulated in cardiomyocytes by ET-1: effects of PD184352. Cardiomyocytes were unstimulated (Control) or exposed to ET-1, PD184352 (PD) or ET-1 in the presence of PD184352 (PD/ET-1). Microarray analysis was performed to identify RNAs significantly upregulated by ET-1 (>1.5-fold change, FDR<0.05) and with significant inhibition or enhancement with PD184352. Raw values are provided for Controls and expression relative to Controls is provided for PD, ET-1 and PD/ET-1. Results are means for 4 separate hybridisations. Where multiple probesets represented the same RNA, individual raw values are provided for controls and, since the relative fold changes were similar, the mean values are provided for the treatments. RNAs in each group (inhibited by PD184352, enhanced by PD184352, no significant effect of PD184352) are listed alphabetically according to gene symbol. (0.51 MB DOC) [file pone.0010027.s001.doc]

**Table S1. RNAs upregulated in cardiomyocytes by ET-1: effects of PD184352.** Cardiomyocytes were unstimulated (Control) or exposed to ET-1, PD184352 (PD) or ET-1 in the presence of PD184352 (PD/ET-1). Microarray analysis was performed to identify RNAs significantly upregulated by ET-1 (>1.5-fold change, FDR<0.05) and with significant inhibition or enhancement with PD184352. Raw values are provided for Controls and expression relative to Controls is provided for PD, ET-1 and PD/ET-1. Results are means for 4 separate hybridisations. Where multiple probesets represented the same RNA, individual raw values are provided for controls and, since the relative fold changes were similar, the mean values are provided for the treatments. RNAs in each group (inhibited by PD184352, enhanced by PD184352, no significant effect of PD184352) are listed alphabetically according to gene symbol.

| **Probeset** | **Gene symbol** | **Classification** | **Control** | **PD** | **ET-1** | **PD/ET-1** |
| --- | --- | --- | --- | --- | --- | --- |
|  |  |  | **(Raw values)** | **(Relative to controls)** | | |
| **Significantly inhibited by PD184352** | | |  |  |  |  |
| 1390881_at | **Abra** | Signaling | 437 | 0.94 | **3.05** | 1.82 |
| 1368223_at | **Adamts1** | Protein synthesis/modification | 1357 | 0.85 | **2.83** | 2.20 |
| 1374610_at | **Agpat9** | Metabolism | 339 | 1.07 | **1.99** | 1.08 |
| 1373843_at, 1382206_a_at, 1385566_at, 1386764_at | **Akap2** | Signaling | 4997, 902, 172, 427 | 0.98 | **1.87** | 1.27 |
| 1372069_at | **Ankrd15** | Signaling | 1959 | 0.90 | **1.54** | 0.99 |
| 1386065_at | **Ankrd57** | Not established | 329 | 0.80 | **2.56** | 1.33 |
| 1367614_at | **Anxa1** | Signaling | 5199 | 1.03 | **1.99** | 1.35 |
| 1387068_at | **Arc** | Not established | 259 | 0.91 | **4.60** | 1.09 |
| 1369871_at | **Areg** | Agonists | 58 | 0.61 | **16.88** | 2.68 |
| 1377750_at | **Arhgef3** | Signaling | 349 | 1.01 | **2.36** | 1.27 |
| 1372964_at, 1382312_at | **Arid5b** | Transcription | 1656, 501 | 1.02 | **2.18** | 1.56 |
| 1376275_at | **Arl5b** | Signaling | 490 | 0.93 | **1.85** | 1.17 |
| 1385961_at | **AS:Klf5** | Potential AS | 117 | 1.23 | **4.50** | 1.70 |
| 1374429_at | **AS:Pim1** | Potential AS | 1386 | 1.27 | **2.27** | 1.64 |
| 1382171_at | **AS:Tsc22d2** | Potential AS | 753 | 0.98 | **2.97** | 1.45 |
| 1385870_at | **AS:Zfp281** | Potential AS | 59 | 0.92 | **2.29** | 1.24 |
| 1395981_at | **Ascc3** | RNA binding/processing | 65 | 1.20 | **1.64** | 0.93 |
| 1369268_at | **Atf3** | Transcription | 453 | 1.72 | **13.68** | 7.37 |
| 1389402_at | **Axud1** | Signaling | 283 | 1.43 | **9.35** | 3.86 |
| 1374433_at | **Bach1** | Transcription | 982 | 0.71 | **1.53** | 0.81 |
| 1373494_at, 1389465_at | **Bcr** | Signaling | 899, 949 | 0.96 | **2.83** | 1.45 |
| 1368677_at | **Bdnf** | Agonists | 155 | 1.00 | **1.63** | 1.26 |
| 1368945_at, 1398270_at | **Bmp2** | Agonists | 142, 472 | 1.00 | **2.08** | 1.21 |
| 1386994_at, 1386995_at | **Btg2** | Transcription | 914, 1100 | 1.14 | **7.70** | 2.85 |
| 1379935_at | **Ccl7** | Agonists | 4224 | 0.68 | **1.93** | 1.48 |
| 1377869_at | **Ccrn4l** | Transcription | 402 | 0.81 | **10.33** | 2.97 |
| 1375910_at | **Cdc42ep3** | Signaling | 3695 | 0.82 | **1.79** | 1.27 |
| 1367601_at, 1367602_at | **Cited2** | Transcription | 2160, 2119 | 1.02 | **4.85** | 3.01 |
| 1385827_at | **Clcf1** | Agonists | 58 | 0.45 | **2.07** | 0.93 |
| 1373866_at | **Coq10b** | Metabolism | 1303 | 1.14 | **2.88** | 2.20 |
| 1390628_at, 1396225_at | **Cpeb2** | RNA binding/processing | 603, 75 | 1.16 | **1.70** | 1.45 |
| 1373525_at, 1379666_at | **Dcun1d3** | Not established | 337, 293 | 0.92 | **1.80** | 1.15 |
| 1388589_at | **Dot1l** | DNA regulation | 687 | 1.06 | **2.57** | 1.52 |
| 1368146_at, 1368147_at | **Dusp1** | Signaling | 1884, 281 | 0.61 | **3.02** | 2.51 |
| 1394028_at | **Dusp10** | Signaling | 192 | 1.00 | **1.94** | 0.97 |
| 1388082_at, 1393119_at | **Dusp4** | Signaling | 113, 1286 | 0.68 | **1.82** | 0.94 |
| 1368124_at | **Dusp5** | Signaling | 473 | 0.57 | **5.02** | 0.90 |
| 1377064_at, 1387024_at | **Dusp6** | Signaling | 1211, 2234 | 0.06 | **2.87** | 0.18 |
| 1369519_at | **Edn1** | Agonists | 293 | 0.94 | **1.51** | 1.10 |
| 1368321_at | **Egr1** | Transcription | 4265 | 0.05 | **4.72** | 0.27 |
| 1387306_a_at, 1398266_a_at | **Egr2** | Transcription | 487, 157 | 0.27 | **7.04** | 0.98 |
| 1369545_at, 1392791_at | **Egr3** | Transcription | 222, 180 | 0.68 | **27.14** | 2.47 |
| 1387442_at | **Egr4** | Transcription | 10 | 1.20 | **72.13** | 2.00 |
| 1374143_at | **Epha2** | Adhesion/ECM | 351 | 0.40 | **1.75** | 0.67 |
| 1369587_at, 1385150_at | **Ereg** | Agonists | 171, 132 | 1.14 | **3.52** | 1.66 |
| 1373093_at | **Errfi1** | Signaling | 1544 | 0.74 | **7.28** | 2.72 |
| 1369182_at | **F3** | Receptors | 151 | 0.96 | **5.59** | 3.08 |
| 1377938_at | **Fam100a** | Not established | 1045 | 0.94 | **1.59** | 1.22 |
| 1383295_at | **Fam110a** | Not established | 165 | 0.97 | **1.82** | 1.03 |
| 1382059_at, 1392747_at | **Fbxo30** | Protein modification | 488, 339 | 0.98 | **1.83** | 1.46 |
| 1368336_at | **Fdx1** | Metabolism | 2218 | 1.02 | **2.09** | 1.12 |
| 1370623_at, 1383516_at, 1386637_at, 1392894_at | **Fgl2** | Adhesion/ECM | 504, 305, 328, 689 | 1.06 | **1.95** | 1.26 |
| 1388496_at | **Flnc** | Cytoskeleton/myofibrillar | 2243 | 1.07 | **1.54** | 1.00 |
| 1373759_at | **FosB** | Transcription | 40 | 4.37 | **151.16** | 39.06 |
| 1368489_at | **Fosl1** | Transcription | 303 | 0.81 | **5.65** | 1.36 |
| 1373035_at, 1387530_a_at | **Fosl2** | Transcription | 1556, 187 | 1.02 | **1.60** | 1.16 |
| 1383222_at | **Frmd6** | Not established | 785 | 1.04 | **1.52** | 1.15 |
| 1372750_at, 1387843_at | **Fst** | Agonists | 454, 358 | 0.87 | **4.18** | 1.30 |
| 1373513_at, 1387221_at | **Gch** | Metabolism | 175, 276 | 0.96 | **1.93** | 1.74 |
| 1370688_at, 1372523_at | **Gclc** | Metabolism | 942, 1155 | 0.97 | **1.58** | 0.97 |
| 1387548_at | **Has2** | Adhesion/ECM | 440 | 0.84 | **7.38** | 3.27 |
| 1368983_at | **Hbegf** | Agonists | 481 | 0.99 | **2.86** | 1.32 |
| 1383519_at | **Hk2** | Metabolism | 945 | 0.86 | **1.57** | 0.97 |
| 1375852_at | **Hmgcr** | Metabolism | 1982 | 0.87 | **1.77** | 1.33 |
| 1370454_at, 1370997_at | **Homer1** | Receptors | 172, 38 | 1.00 | **5.24** | 1.26 |
| 1368247_at, 1370912_at | **Hspa1a/1b** | Protein folding | 335, 432 | 1.16 | **3.17** | 2.48 |
| 1387202_at | **Icam1** | Adhesion/ECM | 2088 | 0.84 | **2.50** | 1.62 |
| 1368878_at, 1388872_at | **Idi1** | Metabolism | 4209, 2059 | 0.94 | **1.68** | 1.25 |
| 1372389_at | **Ier2** | Signaling | 684 | 0.65 | **3.57** | 1.00 |
| 1389355_at, 1389882_at | **Ier5** | Not established | 2153, 116 | 0.84 | **4.16** | 1.87 |
| 1367795_at | **Ifrd1** | Transcription | 1242 | 1.00 | **5.31** | 2.12 |
| 1370692_at, 1387273_at | **Il1rl1** | Receptors | 170, 1127 | 0.98 | **2.63** | 1.16 |
| 1369191_at | **Il6** | Agonists | 131 | 0.93 | **12.87** | 7.10 |
| 1369012_at, 1383486_at | **Inhba** | Agonists | 265, 212 | 0.73 | **5.08** | 1.93 |
| 1367894_at | **Insig1** | Metabolism | 5314 | 0.93 | **1.56** | 1.08 |
| 1379543_at | **Intron:Arhgap10** | Introns | 42 | 0.94 | **2.01** | 0.84 |
| 1380195_at | **Intron:Atp2b1** | Introns | 317 | 1.01 | **1.59** | 1.09 |
| 1381864_at | **Intron:Cacna2d1** | Introns | 72 | 1.16 | **2.38** | 1.52 |
| 1390723_at | **Intron:Ctnna1** | Introns | 243 | 0.79 | **2.71** | 1.09 |
| 1375475_at | **Intron:Dusp5** | Introns | 116 | 0.84 | **5.80** | 0.46 |
| 1397223_at | **Intron:Fgd6** | Introns | 80 | 1.22 | **1.51** | 1.09 |
| 1379106_at | **Intron:Fgrf1** | Introns | 145 | 0.91 | **2.00** | 0.93 |
| 1381343_at | **Intron:Frmd6** | Introns | 218 | 1.10 | **1.61** | 1.06 |
| 1385757_at | **Intron:Homer1** | Introns | 280 | 1.12 | **1.71** | 1.15 |
| 1390671_at | **Intron:Igf1r** | Introns | 179 | 0.89 | **1.73** | 1.25 |
| 1378489_at, 1383828_at | **Intron:Klf14** | Introns | 88, 89 | 1.02 | **4.06** | 1.00 |
| 1396877_at | **Intron:Lamc1** | Introns | 522 | 0.65 | **2.38** | 1.39 |
| 1379089_at, 1380155_at | **Intron:Myh9** | Introns | 501, 850 | 0.94 | **2.34** | 1.91 |
| 1397112_at | **Intron:Pctk2** | Introns | 170 | 0.87 | **2.04** | 1.15 |
| 1393782_at | **Intron:Plagl1** | Introns | 66 | 0.76 | **3.31** | 0.75 |
| 1379477_at | **Intron:Pdcd6ip** | Introns | 144 | 1.13 | **2.00** | 1.37 |
| 1381253_at | **Intron:Slc12a2** | Introns | 140 | 1.14 | **2.12** | 1.61 |
| 1375490_at, 1377212_at | **Intron:Stard13** | Introns | 156, 116 | 0.88 | **1.62** | 0.94 |
| 1378447_at | **Intron:Thrap1** | Introns | 181 | 0.87 | **3.74** | 1.05 |
| 1382036_at | **Intron:Tln1** | Introns | 274 | 0.85 | **1.88** | 1.23 |
| 1398691_at | **Intron:Tsc22d2** | Introns | 92 | 0.96 | **5.38** | 1.95 |
| 1371091_at | **Irs2** | Signaling | 888 | 0.63 | **2.20** | 1.41 |
| 1385649_at | **Itga5** | Adhesion/ECM | 955 | 1.06 | **1.53** | 1.16 |
| 1387788_at | **Junb** | Transcription | 1447 | 0.98 | **1.76** | 1.31 |
| 1368363_at, 1394039_at | **Klf5** | Transcription | 363, 320 | 1.16 | **2.89** | 1.37 |
| 1387060_at, 1388986_at, 1395557_at | **Klf6** | Transcription | 1085, 1896, 592 | 0.66 | **4.33** | 3.29 |
| 1393728_at | **Lif** | Agonists | 333 | 0.64 | **9.73** | 1.46 |
| 1376632_at | **Lmcd1** | Transcription | 1063 | 1.09 | **2.81** | 1.69 |
| 1377610_at | **Lmod2** | Cytoskeleton/myofibrillar | 2287 | 1.09 | **2.61** | 1.80 |
| 1380229_at | **Maff** | Transcription | 289 | 0.89 | **2.22** | 1.28 |
| 1372211_at | **Mafk** | Transcription | 845 | 0.78 | **1.77** | 1.17 |
| 1372520_at, 1373225_at | **Mcl1** | Signaling | 4775, 2687 | 0.86 | **1.58** | 0.98 |
| 1383288_at, 1384427_at | **Mdm2** | Protein modification | 1362, 717 | 1.04 | **1.80** | 1.20 |
| 1378867_at | **Mirn221/222** | Non-protein coding | 85 | 0.62 | **11.42** | 0.92 |
| 1382017_at | **Mirn322/503** | Non-protein coding | 270 | 0.99 | **1.55** | 1.11 |
| 1391643_at | **Mirn224-2/27a/23a** | Non-protein coding | 1163 | 0.66 | **2.63** | 1.01 |
| 1372904_at, 1384900_at | **Mobkl2b** | Signaling | 724, 246 | 0.96 | **1.70** | 1.31 |
| 1368308_at | **Myc** | Transcription | 648 | 0.51 | **3.89** | 1.39 |
| 1370174_at | **Myd116** | Not established | 1424 | 0.97 | **2.26** | 1.89 |
| 1378032_at | **Nfkbiz** | Transcription | 1216 | 1.05 | **3.30** | 2.47 |
| 1369007_at | **Nr4a2** | Transcription | 128, 13 | 1.20 | **18.05** | 8.32 |
| 1369067_at, 1393389_at | **Nr4a3** | Transcription | 126, 119 | 1.76 | **24.68** | 9.24 |
| 1368683_at | **Oldlr1** | Receptors | 1712 | 0.88 | **1.86** | 1.47 |
| 1384254_at | **Otud1** | Protein modification | 1396 | 0.86 | **5.93** | 2.59 |
| 1383224_at | **Pard6b** | Adhesion/ECM | 232 | 1.22 | **3.25** | 1.08 |
| 1385116_at | **Pcdhb21** | Adhesion/ECM | 57 | 1.07 | **1.50** | 1.08 |
| 1390814_at, 1399081_at | **Peli1** | Not established | 910, 537 | 0.94 | **2.01** | 1.71 |
| 1368860_at | **Phlda1** | Signaling | 250 | 0.25 | **5.63** | 0.72 |
| 1396561_x_at | **Piga** | Metabolism | 73 | 1.18 | **1.51** | 1.10 |
| 1387269_s_at | **Plaur** | Receptors | 452 | 0.83 | **2.45** | 1.07 |
| 1368106_at | **Plk2** | Signaling | 2462 | 0.70 | **3.97** | 2.39 |
| 1369029_at | **Plscr1** | Metabolism | 554 | 0.80 | **1.56** | 1.04 |
| 1367841_a_at | **Prl8a9** | Agonists | 52 | 0.96 | **2.33** | 0.80 |
| 1368527_at | **Ptgs2** | Metabolism | 421 | 0.92 | **18.06** | 6.18 |
| 1370193_at | **Ptp4a1** | Signaling | 3227 | 0.96 | **1.54** | 1.15 |
| 1370177_at | **PVR** | Receptors | 1029 | 0.78 | **2.14** | 1.26 |
| 1373730_at | **Rbm33** | RNA binding/processing | 548 | 1.08 | **1.50** | 1.10 |
| 1379714_at, 1381341_at | **rCG_43687** | Not established | 116, 145 | 1.09 | **4.05** | 1.30 |
| 1373679_at | **RGD1306119** | Not established | 469 | 1.01 | **1.90** | 0.83 |
| 1388945_at | **RGD1311307** | Not established | 457 | 0.92 | **1.92** | 1.16 |
| 1383001_at, 1383874_at, 1394278_at | **RGD1560812** | Not established | 236, 333, 287 | 1.00 | **3.13** | 1.07 |
| 1368144_at, 1387074_at | **Rgs2** | Signaling | 955, 1566 | 0.79 | **6.79** | 3.24 |
| 1394527_at | **Ripk2** | Signaling | 163 | 1.03 | **1.54** | 0.92 |
| 1381279_at | **Ripk2** | Signaling | 623 | 0.93 | **2.55** | 1.11 |
| 1381533_at | **Rnd1** | Signaling | 1217 | 1.14 | **2.89** | 1.78 |
| 1376066_at, 1377663_at, 1394077_at | **Rnd3** | Signaling | 1253, 1516, 757 | 0.81 | **1.79** | 1.06 |
| 1371774_at | **Sat** | Metabolism | 3921 | 1.06 | **2.31** | 1.41 |
| 1368487_at | **Serpinb2** | Protein synthesis/modification | 268 | 1.19 | **23.75** | 3.09 |
| 1368519_at, 1392264_s_at | **Serpine1** | Protein synthesis/modification | 377, 163 | 0.93 | **18.58** | 10.51 |
| 1372417_at | **Sertad1** | Transcription | 938 | 1.07 | **1.85** | 1.24 |
| 1367802_at | **Sgk** | Signaling | 1619 | 0.79 | **2.85** | 0.95 |
| 1367853_at | **Slc12a2** | Channels/pumps/transporters | 966 | 1.00 | **1.60** | 1.19 |
| 1370314_at | **Slc20a1** | Channels/pumps/transporters | 2408 | 0.86 | **1.58** | 1.15 |
| 1371754_at | **Slc25a25** | Channels/pumps/transporters | 290 | 0.84 | **5.05** | 3.58 |
| 1368596_at | **Snf1lk** | Signaling | 467 | 0.94 | **2.31** | 1.26 |
| 1374864_at | **Spry2** | Signaling | 1266 | 0.33 | **3.03** | 0.86 |
| 1374829_at, 1388842_at | **Srf** | Transcription | 429, 2731 | 0.91 | **1.85** | 1.43 |
| 1372510_at, 1384331_at | **Srxn1** | Metabolism | 750, 464 | 0.96 | **4.82** | 1.27 |
| 1377340_at | **Tfpi2** | Protein synthesis/modification | 287 | 0.66 | **2.23** | 1.01 |
| 1367859_at | **Tgfb3** | Agonists | 1096 | 0.87 | **1.59** | 1.12 |
| 1368901_at, 1375951_at | **Thbd** | Receptors | 99, 217 | 0.94 | **2.20** | 1.08 |
| 1372926_at, 1375138_at, 1389836_a_at | **Timp3** | Protein synthesis/modification | 553, 1691, 2370 | 0.94 | **1.55** | 1.28 |
| 1374446_at, 1385407_at | **Tiparp** | Protein synthesis/modification | 2268, 1314 | 0.87 | **1.64** | 1.19 |
| 1385641_at | **Tnfaip3** | Transcription | 1045 | 0.93 | **3.08** | 1.46 |
| 1371194_at | **Tnfaip6** | Adhesion/ECM | 723 | 1.18 | **4.39** | 3.26 |
| 1371785_at | **Tnfrsf12a** | Receptors | 4417 | 1.01 | **2.12** | 1.40 |
| 1371019_at | **Trib1** | Signaling | 526 | 0.71 | **2.14** | 0.92 |
| 1384163_at | **Trp53inp1** | Signaling | 1560 | 0.95 | **1.54** | 1.19 |
| 1375031_at | **Ttn** | Cytoskeleton/myofibrillar | 338 | 1.13 | **1.56** | 1.04 |
| 1378165_at, 1387750_at | **Twist1** | Transcription | 445, 103 | 0.90 | **3.83** | 1.00 |
| 1379910_at | **Uap1** | Metabolism | 1091 | 0.92 | **2.87** | 1.35 |
| 1390248_at | **Unknown** | Unknown | 132 | 1.19 | **1.59** | 1.05 |
| 1395119_at | **Unknown** | Unknown | 281 | 0.95 | **1.70** | 0.95 |
| 1395168_at | **Unknown** | Unknown | 140 | 1.13 | **1.56** | 1.15 |
| 1376403_at | **Unknown** | Unknown | 199 | 1.05 | **2.84** | 1.23 |
| 1378754_at | **Unknown** | Unknown | 429 | 1.21 | **2.12** | 1.51 |
| 1392108_at | **Unknown** | Unknown | 94 | 1.11 | **3.39** | 1.88 |
| 1373651_at | **Unknown** | Unknown | 425 | 0.64 | **1.95** | 0.95 |
| 1379584_at | **Unknown** | Unknown | 259 | 0.61 | **1.86** | 1.06 |
| 1385578_at | **Utp14b** | RNA binding/processing | 180 | 1.27 | **1.78** | 1.16 |
| 1377778_at | **Vof16** | Not established | 302 | 0.87 | **3.17** | 1.28 |
| 1389119_at | **Xirp1** | Cytoskeleton/myofibrillar | 2408 | 0.88 | **1.82** | 0.98 |
| 1373767_at | **Zfand2a** | Not established | 746 | 0.96 | **1.62** | 1.01 |
| 1375130_at, 1383905_at, 1388868_at | **Zfand5** | Transcription | 657, 469, 3626 | 0.99 | **1.81** | 1.18 |
| 1382642_at, 1385869_at | **Zfp281** | Transcription | 1054, 977 | 0.79 | **2.45** | 1.32 |
| 1387870_at | **Zfp36** | RNA binding/processing | 1447 | 0.90 | **2.86** | 1.76 |
| 1373106_at | **Zfp36l2** | RNA binding/processing | 1966 | 1.17 | **1.78** | 1.23 |
| 1373680_at | **Znf697** | Transcription | 343 | 0.86 | **2.35** | 1.11 |
| 1388130_at | **Zyx** | Cytoskeleton/myofibrillar | 5625 | 0.96 | **1.56** | 1.27 |
|  |  |  |  |  |  |  |
| **Significantly enhanced by PD184352** | | |  |  |  |  |
| 1369788_s_at, 1374404_at, 1389528_s_at | **Jun** | Transcription | 853, 511, 1429 | 1.29 | **2.77** | 5.38 |
| 1375248_at, 1376569_at, 1386041_a_at, 1394068_x_at | **Klf2** | Transcription | 812, 648, 134, 210 | 1.76 | **2.66** | 5.91 |
| 1373403_at | **LOC684871** | Not established | 92 | 2.93 | **3.30** | 5.03 |
| 1376937_at | **RGD1565927** | Not established | 1083 | 1.00 | **1.77** | 2.40 |
| 1369958_at | **Rhob** | Signaling | 2621 | 1.30 | **2.89** | 4.14 |
| 1370287_a_at, 1379936_at, 1395794_at | **Tpm1** | Cytoskeleton/myofibrillar | 2294, 2137, 1431 | 1.04 | **1.53** | 1.79 |
|  |  |  |  |  |  |  |
| **No significant effect of PD184352** | | |  |  |  |  |
| 1379311_at, 1380772_at, 1397437_at | **Arid5a** | Transcription | 407, 184, 273 | 1.09 | **2.03** | 1.78 |
| 1390850_at | **AS: Adfp** | Potential AS sequences | 100 | 1.44 | **2.23** | 1.32 |
| 1398037_at | **AS: Plk2** | Potential AS sequences | 111 | 0.96 | **2.12** | 1.55 |
| 1386062_at | **AS: Rnd3** | Potential AS sequences | 69 | 1.34 | **2.53** | 1.45 |
| 1384659_at | **AS: Sfp295** | Potential AS sequences | 39 | 1.39 | **1.91** | 2.24 |
| 1375538_at | **AS: Vcl** | Potential AS sequences | 4828 | 1.27 | **1.70** | 1.54 |
| 1369807_at | **Bdkrb1** | Receptors | 104 | 1.68 | **2.26** | 1.76 |
| 1368050_at | **Ccnl1** | Signaling | 953 | 1.12 | **2.21** | 2.32 |
| 1380063_at | **Ch25h** | Metabolism | 590 | 0.42 | **8.86** | 10.94 |
| 1389573_at | **Chac1** | Channels/pumps/transporters | 1060 | 1.25 | **2.55** | 2.36 |
| 1389368_at | **Cnksr3** | Not established | 790 | 1.25 | **1.67** | 1.38 |
| 1369737_at, 1378925_at, 1387714_at, 1393550_at | **Crem** | Transcription | 114, 362, 116, 99 | 1.15 | **1.97** | 1.80 |
| 1367631_at | **Ctgf** | Agonists | 12573 | 0.80 | **1.90** | 1.95 |
| 1368290_at | **Cyr61** | Adhesion/ECM | 711 | 0.74 | **9.32** | 9.45 |
| 1372722_at | **Dnajb4** | Protein synthesis/modification | 1288 | 1.07 | **2.27** | 2.17 |
| 1372385_at | **Dusp8** | Signaling | 387 | 1.16 | **1.52** | 1.88 |
| 1385251_at | **Fam110c** | Cytoskeleton/myofibrillar | 78 | 1.15 | **3.35** | 2.81 |
| 1375043_at | **Fos** | Transcription | 238 | 0.84 | **14.99** | 9.16 |
| 1388792_at | **Gadd45g** | Signaling | 1816 | 1.09 | **2.42** | 2.09 |
| 1374903_at | **Gcnt2** | Protein synthesis/modification | 291 | 0.92 | **1.56** | 1.32 |
| 1382351_at | **Gem** | Signaling | 384 | 1.17 | **2.06** | 2.09 |
| 1369113_at | **Grem1** | Agonists | 144 | 0.95 | **1.57** | 1.41 |
| 1394022_at | **Id4** | Transcription | 654 | 1.02 | **1.63** | 1.67 |
| 1388587_at | **Ier3** | Signaling | 5086 | 0.78 | **1.58** | 1.62 |
| 1396539_at | **Intron:Actn1** | Introns | 247 | 0.93 | **5.63** | 4.23 |
| 1391753_at | **Intron:Ankrd1** | Introns | 1012 | 0.40 | **1.82** | 1.43 |
| 1394451_at | **Intron:Anxa1** | Introns | 432 | 0.96 | **2.81** | 2.22 |
| 1392250_at | **Intron:Anxa2** | Introns | 96 | 1.13 | **2.07** | 1.39 |
| 1397030_at | **Intron:Atp2b1** | Introns | 104 | 0.96 | **2.02** | 1.27 |
| 1396834_at | **Intron:Braf** | Introns | 79 | 1.44 | **1.52** | 1.45 |
| 1390618_at | **Intron:Creb5a** | Introns | 119 | 1.25 | **1.77** | 1.47 |
| 1397449_at | **Intron:Enah** | Introns | 378 | 0.69 | **1.97** | 1.75 |
| 1391373_at | **Intron:Enah** | Introns | 206 | 0.93 | **1.87** | 1.62 |
| 1394750_at | **Intron:Fhl1** | Introns | 171 | 0.96 | **2.32** | 2.02 |
| 1380230_at | **Intron:Gch** | Introns | 57 | 1.08 | **1.86** | 2.18 |
| 1382982_at | **Intron:Gnb1** | Introns | 200 | 0.99 | **1.67** | 1.37 |
| 1381651_at | **Intron:March7** | Introns | 385 | 1.11 | **1.63** | 1.51 |
| 1380311_at | **Intron:Ppp1r12a** | Introns | 186 | 1.13 | **2.28** | 1.85 |
| 1381430_at | **Intron:Rheb** | Introns | 37 | 0.55 | **2.34** | 1.76 |
| 1391215_at | **Intron:Samd4** | Introns | 227 | 0.94 | **1.85** | 1.68 |
| 1380701_at | **Intron:Ssfa2** | Introns | 260 | 0.94 | **1.51** | 1.17 |
| 1397115_at | **Intron:Tbc1d1** | Introns | 86 | 1.61 | **2.06** | 1.50 |
| 1373623_at | **Itpkc** | Signaling | 355 | 1.27 | **1.68** | 1.35 |
| 1387260_at | **Klf4** | Transcription | 1339 | 1.13 | **3.42** | 2.98 |
| 1376700_at | **Lima1** | Cytoskeleton/myofibrillar | 1391 | 0.97 | **1.72** | 1.76 |
| 1381798_at | **Lmo7** | Adhesion/ECM | 747 | 0.88 | **1.51** | 1.22 |
| 1371350_at, 1387737_at | **Mat2a** | Metabolism | 3636, 1288 | 1.03 | **1.80** | 1.65 |
| 1367796_at | **Mgat1** | Protein modification | 1004 | 0.99 | **1.52** | 1.46 |
| 1385028_at | **Mirhg1** | Non-protein coding | 108 | 0.94 | **1.95** | 1.48 |
| 1374290_at, 1396435_at | **Mirn143/145** | Non-protein coding | 338, 192 | 0.98 | **2.17** | 1.91 |
| 1398020_at | **Mirn23b** | Non-protein coding | 268 | 0.92 | **1.67** | 1.84 |
| 1371692_at | **Mllt11** | Not established | 1539 | 0.92 | **1.54** | 1.17 |
| 1371595_at, 1397164_at | **Neat1** | Non-protein-coding | 2697, 771 | 0.99 | **2.17** | 2.01 |
| 1368488_at | **Nfil3** | Transcription | 700 | 1.15 | **3.09** | 3.19 |
| 1374767_at | **Npepo** | Protein synthesis/modification | 1116 | 0.99 | **1.74** | 1.50 |
| 1386935_at | **Nr4a1** | Transcription | 578 | 1.87 | **19.01** | 15.32 |
| 1389918_at | **Palld** | Cytoskeleton/myofibrillar | 847 | 0.99 | **2.04** | 1.48 |
| 1385229_at | **Pcdh20** | Adhesion/ECM | 123 | 1.15 | **1.64** | 1.49 |
| 1368303_at | **Per2** | Transcription | 295 | 1.17 | **1.80** | 1.47 |
| 1369862_at | **Pim1** | Signaling | 88 | 1.15 | **1.78** | 1.37 |
| 1376724_at | **Prkab2** | Signaling | 261 | 1.06 | **1.66** | 1.58 |
| 1383322_at | **Rasl11b** | Signaling | 1218 | 1.07 | **2.50** | 2.78 |
| 1373989_at, 1392688_at | **Rassf1** | Signaling | 696, 126 | 0.94 | **1.77** | 1.55 |
| 1381923_at | **RGD1564664** | Not established | 349 | 1.23 | **2.24** | 2.01 |
| 1389258_at | **Rnf138** | Protein synthesis/modification | 268 | 0.92 | **1.51** | 1.19 |
| 1382500_at, 1391607_at | **Sesn2** | Metabolism | 673, 1034 | 1.05 | **1.56** | 1.46 |
| 1387408_at | **Siah2** | Protein synthesis/modification | 182 | 1.14 | **1.88** | 1.45 |
| 1372347_at | **Skil** | Transcription | 2020 | 1.06 | **1.63** | 1.46 |
| 1377404_at, 1387623_at, 1393559_at, 1396101_at | **Stc1** | Agonists | 1948, 389, 1794, 1231 | 0.91 | **1.51** | 1.82 |
| 1367570_at | **Tagln** | Cytoskeleton/myofibrillar | 3236 | 1.01 | **1.55** | 1.55 |
| 1374529_at, 1394109_at | **Thbs1** | Adhesion/ECM | 8617, 4256 | 0.90 | **3.14** | 2.62 |
| 1383005_at | **Tmem49** | Not established | 730 | 0.77 | **1.65** | 1.43 |
| 1373401_at | **Tnc** | Adhesion/ECM | 933 | 1.19 | **2.49** | 1.73 |
| 1369407_at | **Tnfrsf11b** | Receptors | 1498 | 0.94 | **1.90** | 2.17 |
| 1381070_at | **Tslp** | Agonists | 171 | 0.92 | **1.50** | 1.45 |
| 1384892_at | **Unknown** | Unknown | 139 | 1.11 | **1.85** | 1.45 |
| 1380223_at | **Unknown** | Unknown | 58 | 1.50 | **1.63** | 1.19 |
| 1398059_at | **Unknown** | Unknown | 83 | 1.34 | **1.52** | 1.43 |
| 1380399_at | **Unknown** | Unknown | 220 | 0.97 | **1.51** | 0.99 |
| 1397283_at | **Unknown** | Unknown | 146 | 1.26 | **1.79** | 1.65 |
| 1372905_at, 1398476_at | **Vcl** | Cytoskeleton/myofibrillar | 3414, 930 | 1.09 | **1.62** | 1.34 |
| 1384709_at | **Vgll3** | Transcription | 167 | 0.90 | **1.50** | 1.42 |
| 1395896_at | **Zfp295** | Transcription | 124 | 1.19 | **1.75** | 1.81 |
