# Supplementary material for: ERK1/2 Signaling Dominates Over RhoA Signaling in Regulating Early Changes in RNA Expression Induced by Endothelin-1 in Neonatal Rat Cardiomyocytes
Source: PLoS One. 2010 Apr 2;5(4):e10027. doi: 10.1371/journal.pone.0010027 (PMC2848868; doi:10.1371/journal.pone.0010027)
Supplement: Table S2 — RNAs downregulated in cardiomyocytes by ET-1: effects of PD184352. Cardiomyocytes were unstimulated (Control) or exposed to ET-1, PD184352 (PD) or ET-1 in the presence of PD184352 (PD/ET-1). Microarray analysis was performed to identify RNAs significantly downregulated by ET-1 (>1.5-fold change, FDR<0.05) and with significant inhibition or enhancement with PD184352. Raw values are provided for Controls and expression relative to Controls is provided for PD, ET-1 and PD/ET-1. Results are means for 4 separate hybridisations. Where multiple probesets represented the same RNA, individual raw values are provided for controls and, since the relative fold changes were similar, the mean values are provided for the treatments. RNAs in each group (inhibited by PD184352, no significant effect of PD184352) are listed alphabetically according to gene symbol. (0.23 MB DOC) [file pone.0010027.s002.doc]

**Table S2. RNAs downregulated in cardiomyocytes by ET-1: effects of PD184352.** Cardiomyocytes were unstimulated (Control) or exposed to ET-1, PD184352 (PD) or ET-1 in the presence of PD184352 (PD/ET-1). Microarray analysis was performed to identify RNAs significantly downregulated by ET-1 (>1.5-fold change, FDR<0.05) and with significant inhibition or enhancement with PD184352. Raw values are provided for Controls and expression relative to Controls is provided for PD, ET-1 and PD/ET-1. Results are means for 4 separate hybridisations. Where multiple probesets represented the same RNA, individual raw values are provided for controls and, since the relative fold changes were similar, the mean values are provided for the treatments. RNAs in each group (inhibited by PD184352, no significant effect of PD184352) are listed alphabetically according to gene symbol.

| **Probeset** | **Gene symbol** | **Classification** | **Control** | **PD** | **ET-1** | **PD/ET-1** |
| --- | --- | --- | --- | --- | --- | --- |
|  |  |  | **(Raw values)** | **(Relative to controls)** | | |
| **Significant inhibition by PD184352** | | |  |  |  |  |
| 1390931_at | **Adamts15** | Protein synthesis/modification | 1070 | 1.17 | **0.54** | 0.67 |
| 1383848_at | **Adrb1** | Receptors | 627 | 1.02 | **0.44** | 0.66 |
| 1389264_at | **Ankrd54** | Not established | 376 | 0.86 | **0.57** | 0.83 |
| 1373287_at | **Atoh8** | Transcription | 863 | 1.25 | **0.43** | 0.88 |
| 1370823_at | **Bambi** | Receptors | 960 | 1.33 | **0.62** | 1.00 |
| 1385627_at, 1398482_at | **Bcl3** | Transcription | 652, 503 | 1.29 | **0.48** | 0.61 |
| 1384214_a_at | **Ccnt2** | Transcription | 211 | 0.92 | **0.54** | 0.93 |
| 1368813_at, 1387343_at | **Cebpd** | Transcription | 1479, 2974 | 0.79 | **0.42** | 0.92 |
| 1368025_at | **Ddit4** | Signaling | 1910 | 1.34 | **0.30** | 0.76 |
| 1376612_at | **Dtwd1** | Not established | 252 | 1.10 | **0.65** | 0.93 |
| 1373114_at | **Dtx4** | Signaling | 1566 | 0.95 | **0.65** | 0.75 |
| 1393058_at | **Eid2** | Transcription | 490 | 0.97 | **0.62** | 0.93 |
| 1374530_at | **Fzd7** | Receptors | 339 | 1.06 | **0.66** | 0.98 |
| 1374234_at | **Hkr3** | Transcription | 246 | 1.01 | **0.66** | 0.88 |
| 1389675_at, 1391026_at | **Ier5l** | Not established | 999, 1430 | 1.29 | **0.32** | 0.45 |
| 1378945_at | **Intron:Aco2** | Introns | 241 | 0.86 | **0.58** | 0.84 |
| 1396420_at | **Intron:Baz1b** | Introns | 170 | 0.93 | **0.64** | 1.02 |
| 1385506_at | **Intron:Cdon** | Introns | 298 | 1.32 | **0.58** | 0.81 |
| 1392650_at | **Intron:Csnk2a1** | Introns | 320 | 0.99 | **0.63** | 0.86 |
| 1398560_at | **Intron:Dscr1l1** | Introns | 145 | 1.01 | **0.41** | 0.80 |
| 1376522_at | **Intron:Fabp3** | Introns | 318 | 1.20 | **0.46** | 0.76 |
| 1393540_at | **Intron:Myh6** | Introns | 512 | 0.96 | **0.62** | 0.78 |
| 1393406_at | **Intron:Rbx1** | Introns | 311 | 0.96 | **0.65** | 0.95 |
| 1392317_at | **Intron:Scl33a1** | Introns | 121 | 1.03 | **0.49** | 0.76 |
| 1368073_at | **Irf1** | Transcription | 907 | 1.12 | **0.41** | 0.72 |
| 1368249_at, 1381396_s_at | **Klf15** | Transcription | 931, 288 | 1.01 | **0.40** | 0.76 |
| 1378332_at, 1389479_at, 1393150_at | **Klf3** | Transcription | 1254, 1744, 1242 | 0.98 | **0.60** | 0.82 |
| 1381065_at | **LOC499120** | Not established | 670 | 0.92 | **0.64** | 1.02 |
| 1381267_at | **LOC691468** | Not established | 138 | 0.92 | **0.57** | 1.00 |
| 1372060_at | **Lysmd4** | Not established | 413 | 0.90 | **0.56** | 0.70 |
| 1376718_at | **Mblac1** | Not established | 499 | 0.97 | **0.61** | 1.00 |
| 1396481_at | **Mirn1-2/-133a-1** | Non-protein coding | 75 | 0.47 | **0.34** | 1.28 |
| 1388525_at | **Pik3ip1** | Signaling | 1024 | 1.26 | **0.53** | 0.75 |
| 1378484_at | **Rasl12** | Signaling | 684 | 0.92 | **0.58** | 0.76 |
| 1385253_at | **RGD1304610** | Not established | 173 | 1.07 | **0.64** | 0.95 |
| 1374364_at | **RGD1562335** | Not established | 392 | 0.98 | **0.51** | 0.77 |
| 1374756_at | **RGD1562665** | Not established | 181 | 1.05 | **0.35** | 0.84 |
| 1390249_at | **Sept14** | Signaling | 503 | 0.87 | **0.65** | 0.91 |
| 1375121_at, 1394025_at | **Smad6** | Transcription | 944, 650 | 1.06 | **0.63** | 0.97 |
| 1393262_at | **Smad9** | Transcription | 454 | 1.12 | **0.65** | 1.02 |
| 1373219_at | **Snai1** | Transcription | 1379 | 1.05 | **0.27** | 0.37 |
| 1397220_at | **Snhg7** | Non-protein-coding | 657 | 0.93 | **0.64** | 0.99 |
| 1372633_at | **Spg20** | Trafficking | 684 | 0.97 | **0.64** | 0.81 |
| 1390215_at | **Ston2** | Trafficking | 127 | 1.20 | **0.64** | 1.02 |
| 1383282_at | **Thap11** | DNA regulation | 566 | 1.08 | **0.65** | 0.94 |
| 1390928_at | **Tigd2** | DNA regulation | 267 | 0.87 | **0.41** | 0.79 |
| 1391027_at | **Trim65** | Not established | 370 | 0.90 | **0.58** | 0.81 |
| 1371131_a_at | **Txnip** | Transcription | 5283 | 1.31 | **0.65** | 0.77 |
| 1389419_at | **Unknown** | Unknown | 576 | 0.87 | **0.55** | 0.90 |
| 1389714_at | **Unknown** | Unknown | 201 | 0.91 | **0.65** | 0.85 |
| 1381619_at | **Unknown** | Unknown | 131 | 1.06 | **0.38** | 0.91 |
| 1383977_a_at | **Unknown** | Unknown | 152 | 1.05 | **0.37** | 0.71 |
| 1389084_at | **Unknown** | Unknown | 377 | 0.99 | **0.47** | 0.67 |
| 1378117_at | **Unknown** | Unknown | 317 | 1.17 | **0.64** | 0.94 |
| 1393512_at | **Unknown** | Unknown | 192 | 1.15 | **0.63** | 0.85 |
| 1383058_at | **Unknown** | Unknown | 2498 | 1.29 | **0.45** | 0.83 |
| 1391863_at | **Unknown** | Unknown | 348 | 1.30 | **0.18** | 0.56 |
| 1380914_at, 1393652_at | **Zbtb1** | Transcription | 209, 534 | 1.04 | **0.64** | 0.87 |
| 1377154_at | **Zfp157** | Transcription | 251 | 1.04 | **0.58** | 0.80 |
| 1372205_at | **Zfp278** | Transcription | 417 | 1.12 | **0.66** | 0.88 |
| 1393438_at | **Zfp316** | Transcription | 203 | 1.20 | **0.62** | 0.93 |
| 1370984_at | **Zfp46** | Transcription | 660 | 1.23 | **0.66** | 0.85 |
| 1386793_at | **Zfp61** | Transcription | 183 | 1.16 | **0.42** | 0.79 |
| 1381282_at | **Zfp768** | Transcription | 277 | 1.04 | **0.64** | 0.88 |
| 1386633_at | **Zfp869** | Transcription | 276 | 0.89 | **0.60** | 0.89 |
| 1385597_at | **Znf124** | Transcription | 83 | 1.24 | **0.39** | 0.68 |
| 1389366_at | **Znf553** | Transcription | 432 | 1.06 | **0.51** | 0.75 |
| 1372699_at | **Znf775** | Transcription | 201 | 0.97 | **0.54** | 0.74 |
| 1384432_at | **Zscan12** | Transcription | 199 | 1.07 | **0.62** | 0.89 |
|  |  |  |  |  |  |  |
| **Not significantly inhibited by PD184352** | | |  |  |  |  |
| 1390974_a_at | **Adat2** | RNA binding/processing | 392 | 0.85 | **0.61** | 0.88 |
| 1381153_at | **Anapc4** | Protein synthesis/modification | 284 | 0.86 | **0.58** | 0.78 |
| 1374493_at | **Bmf** | Signaling | 424 | 1.17 | **0.63** | 0.71 |
| 1389145_at | **Cdc42ep2** | Signaling | 635 | 0.91 | **0.61** | 0.70 |
| 1390024_at | **Clec2d/g** | Receptors | 1686 | 0.81 | **0.64** | 0.61 |
| 1398710_at | **Cyp2u1** | Metabolism | 427 | 0.95 | **0.58** | 0.73 |
| 1383205_at | **Dact2** | Not established | 186 | 1.15 | **0.66** | 0.76 |
| 1391741_a_at | **Fam78a** | Not established | 338 | 0.94 | **0.63** | 0.73 |
| 1390995_at | **Fkbpl** | Protein synthesis/modification | 316 | 1.08 | **0.65** | 0.79 |
| 1389404_at | **Foxs1** | Transcription | 553 | 0.58 | **0.54** | 0.58 |
| 1389871_at | **Got2** | Metabolism | 91 | 0.90 | **0.63** | 0.90 |
| 1387036_at | **Hes1** | Transcription | 1576 | 1.12 | **0.58** | 0.54 |
| 1387028_a_at | **Id1** | Transcription | 3595 | 0.84 | **0.61** | 0.64 |
| 1380168_at | **Intron:Etv4** | Introns | 140 | 0.64 | **0.66** | 0.75 |
| 1391171_at | **Intron:Itm2b** | Introns | 303 | 1.18 | **0.59** | 0.63 |
| 1392578_at | **Intron:Mgp** | Introns | 626 | 0.49 | **0.21** | 0.10 |
| 1397165_at | **Intron:Smarca2** | Introns | 148 | 0.77 | **0.62** | 0.63 |
| 1374786_at | **Intron:Tia1** | Introns | 151 | 1.08 | **0.49** | 0.74 |
| 1390776_at, 1394251_x_at | **Irx3** | Transcription | 1232, 566 | 0.49 | **0.66** | 0.48 |
| 1390969_at | **Kcne4** | Channels/pumps/transporters | 1275 | 0.92 | **0.62** | 0.74 |
| 1389988_at | **Kctd2** | Channels/pumps/transporters | 989 | 0.93 | **0.67** | 0.79 |
| 1385431_at | **LOC688018** | Signaling | 250 | 0.78 | **0.63** | 0.78 |
| 1374693_at | **Parp16** | Metabolism | 459 | 0.95 | **0.55** | 0.63 |
| 1384262_at | **Ppp1r3b** | Signaling | 487 | 0.90 | **0.49** | 0.62 |
| 1384937_at | **Rbak** | Transcription | 112 | 1.32 | **0.53** | 0.76 |
| 1380314_at | **RGD1563072** | Not established | 280 | 0.99 | **0.66** | 0.85 |
| 1392170_at | **Rwdd3** | Not established | 180 | 0.91 | **0.66** | 0.74 |
| 1367920_at | **S1pr2** | Receptors | 622 | 0.90 | **0.61** | 0.70 |
| 1397522_at | **Sbf1** | Signaling | 315 | 0.76 | **0.65** | 0.62 |
| 1383589_at | **Snai2** | Transcription | 515 | 1.16 | **0.62** | 0.84 |
| 1369584_at, 1377092_at | **Socs3** | Signaling | 732, 2404 | 1.05 | **0.56** | 0.67 |
| 1388786_at | **Synpo** | Cytoskeleton/myofibrillar | 2163 | 0.99 | **0.66** | 0.75 |
| 1393153_at | **Tardbp** | RNA binding/processing | 234 | 1.17 | **0.66** | 0.90 |
| 1397692_at | **Tia1** | Protein synthesis/modification | 133 | 1.02 | **0.52** | 0.73 |
| 1393649_at | **Tmpo** | Transcription | 102 | 1.19 | **0.55** | 0.62 |
| 1397527_at | **Trim47** | Not established | 784 | 0.89 | **0.54** | 0.74 |
| 1378547_at | **Unknown** | Unknown | 437 | 1.00 | **0.66** | 0.79 |
| 1377629_at | **Unknown** | Unknown | 380 | 1.01 | **0.65** | 0.77 |
| 1379454_at | **Unknown** | Unknown | 150 | 1.10 | **0.65** | 0.77 |
| 1383919_at | **Unknown** | Unknown | 154 | 0.81 | **0.64** | 0.74 |
| 1381077_at | **Unknown** | Unknown | 176 | 0.91 | **0.63** | 0.79 |
| 1392605_at | **Unknown** | Unknown | 181 | 0.94 | **0.62** | 0.88 |
| 1378367_at | **Unknown** | Unknown | 143 | 1.43 | **0.61** | 0.77 |
| 1377045_at | **Unknown** | Unknown | 286 | 0.88 | **0.58** | 0.59 |
| 1376358_at | **Unknown** | Unknown | 98 | 1.19 | **0.45** | 0.76 |
| 1391507_at | **Znf467** | Transcription | 385 | 1.06 | **0.60** | 0.62 |
