# Supplementary material for: ERK1/2 Signaling Dominates Over RhoA Signaling in Regulating Early Changes in RNA Expression Induced by Endothelin-1 in Neonatal Rat Cardiomyocytes
Source: PLoS One. 2010 Apr 2;5(4):e10027. doi: 10.1371/journal.pone.0010027 (PMC2848868; doi:10.1371/journal.pone.0010027)
Supplement: Table S3 — RNAs regulated in cardiomyocytes by PD184352 alone or PD184352 in the presence of ET-1. Cardiomyocytes were unstimulated (Control) or exposed to ET-1, PD184352 (PD) or ET-1 in the presence of PD184352 (PD/ET-1). Microarray analysis was performed. RNAs not significantly regulated by ET-1 alone but significantly regulated by PD or PD/ET-1 were selected (>1.5-fold change, FDR<0.05) and clustered according to upregulation or downregulation. Raw values are provided for Controls and expression relative to Controls is provided for PD, ET-1 and PD/ET-1. Results are means for 4 separate hybridisations. Where multiple probesets represented the same RNA, individual raw values are provided for controls and, since the relative fold changes were similar, the mean values are provided for the treatments. RNAs in each group are listed alphabetically according to gene symbol. (0.15 MB DOC) [file pone.0010027.s003.doc]

**Table S3. RNAs regulated in cardiomyocytes by PD184352 alone or PD184352 in the presence of ET-1.** Cardiomyocytes were unstimulated (Control) or exposed to ET-1, PD184352 (PD) or ET-1 in the presence of PD184352 (PD/ET-1). Microarray analysis was performed. RNAs not significantly regulated by ET-1 alone but significantly regulated by PD or PD/ET-1 were selected (>1.5-fold change, FDR<0.05) and clustered according to upregulation or downregulation. Raw values are provided for Controls and expression relative to Controls is provided for PD, ET-1 and PD/ET-1. Results are means for 4 separate hybridisations. Where multiple probesets represented the same RNA, individual raw values are provided for controls and, since the relative fold changes were similar, the mean values are provided for the treatments. RNAs in each group are listed alphabetically according to gene symbol.

| **Probeset** | **Gene symbol** | **Classification** | **Control** | **PD** | **ET-1** | **PD/ET-1** |
| --- | --- | --- | --- | --- | --- | --- |
|  |  |  | **(Raw values)** | **(Relative to controls)** | | |
| **PD184352 upregulation of baseline RNA expression** | | |  |  |  |  |
| 1382413_at | **AS:Nup155** | Potential AS | 63 | **1.66** | 1.13 | 1.14 |
| 1385706_at | **AS:Tes** | Potential AS | 77 | **1.53** | 1.41 | 1.19 |
| 1387713_a_at | **Fcer1a** | Receptors | 57 | **1.51** | 1.17 | 0.89 |
| 1372016_at | **Gadd45b** | Signaling | 618 | **1.64** | 1.37 | 1.88 |
| 1396919_at | **Intron:Prkwnk1** | Introns | 42 | **2.10** | 1.44 | 1.40 |
| 1385177_at | **Intron:Sorbs1** | Introns | 100 | **1.51** | 1.50 | 1.29 |
| 1379914_at | **Klf11** | Transcriptional regulation | 391 | **1.78** | 0.76 | 1.49 |
| 1378074_at | **Pdk4** | Regulation of metabolism | 330 | **1.62** | 0.99 | 1.01 |
| 1381497_at | **Unknown** | Unknown | 30 | **2.42** | 1.70 | 1.58 |
| 1380925_at | **Unknown** | Unknown | 66 | **1.73** | 0.87 | 1.33 |
| 1383029_at | **Unknown** | Unknown | 43 | **1.73** | 1.41 | 1.01 |
| 1396637_at | **Unknown** | Unknown | 69 | **1.64** | 1.36 | 1.08 |
|  |  |  |  |  |  |  |
| **PD184352 downregulation of baseline RNA expression** | | |  |  |  |  |
| 1382778_at | **AS:Dusp6** | Potential AS | 567 | **0.03** | 2.07 | 0.12 |
| 1369415_at | **Bhlhb2** | Transcriptional regulation | 1247 | **0.63** | 1.06 | 0.69 |
| 1369815_at | **Ccl3** | Agonists | 939 | **0.65** | 0.97 | 0.62 |
| 1369529_at | **Csf3** | Agonists | 152 | **0.37** | 0.72 | 1.07 |
| 1387316_at | **Cxcl1** | Agonists | 6292 | **0.64** | 1.40 | 1.01 |
| 1368760_at | **Cxcl2** | Agonists | 576 | **0.48** | 1.31 | 0.58 |
| 1389894_at | **Dlc1** | Signaling | 210 | **0.55** | 0.80 | 0.76 |
| 1379790_at | **Dll4** | Agonists | 435 | **0.55** | 0.95 | 0.67 |
| 1387270_at | **Hhex** | Transcriptional regulation | 283 | **0.46** | 0.84 | 0.48 |
| 1368592_at, 1371170_a_at | **Il1a** | Agonists | 371, 283 | **0.65** | 0.98 | 0.54 |
| 1392024_at | **Intron:Spred1** | Introns | 128 | **0.43** | 1.08 | 0.66 |
| 1376648_at | **Mycn** | Transcriptional regulation | 356 | **0.64** | 0.83 | 0.81 |
| 1382138_at | **Nrarp** | Not established | 323 | **0.60** | 1.05 | 0.59 |
| 1398287_at | **Plau** | Protein synthesis/modification | 323 | **0.64** | 0.92 | 0.65 |
| 1393638_at | **Ptger4** | Receptors | 632 | **0.48** | 1.05 | 0.75 |
| 1373777_at | **Rgs16** | Signaling | 547 | **0.64** | 1.39 | 0.93 |
| 1392529_at | **Spry4** | Signaling | 1421 | **0.41** | 1.22 | 0.58 |
| 1391384_at | **Tnf** | Agonists | 122 | **0.20** | 0.88 | 0.09 |
| 1388821_at | **Trib2** | Signaling | 2240 | **0.67** | 0.99 | 0.94 |
| 1376426_at | **Unknown** | Unknown | 93 | **0.61** | 1.13 | 1.09 |
| 1379141_at | **Unknown** | Unknown | 82 | **0.58** | 1.12 | 1.00 |
| 1392788_at | **Unknown** | Unknown | 240 | **0.34** | 1.25 | 0.55 |
|  |  |  |  |  |  |  |
| **PD184352/ET-1 upregulation of baseline RNA expression** | | |  |  |  |  |
| 1389546_at | **Amotl2** | Adhesion/ECM | 3427 | 0.96 | 1.26 | **1.70** |
| 1385925_at | **AS:Glipr2** | Potential AS | 1269 | 1.08 | 1.13 | **1.58** |
| 1394940_at | **Fam46a** | Not established | 2033 | 0.89 | 1.30 | **1.53** |
| 1397197_at | **Intron:SerinC3** | Introns | 139 | 1.08 | 1.31 | **1.53** |
| 1390471_at, 1391841_at, 1395350_at | **Intron:Tpm1** | Introns | 768, 1869, 311 | 1.04 | 1.38 | **1.61** |
| 1378081_at | **Intron:Tsc22d1** | Introns | 254 | 1.15 | 1.46 | **1.54** |
| 1387264_at | **Kcnk6** | Channels/pumps/transporters | 198 | 1.02 | 1.35 | **2.02** |
| 1369393_at | **Map3k8** | Signaling | 219 | 1.39 | 0.82 | **1.95** |
| 1380682_at | **Mex3b** | RNA processing | 345 | 1.16 | 1.15 | **1.91** |
| 1374650_at, 1396053_at | **Nedd9** | Cytoskeleton/myofibrillar | 625, 262 | 1.20 | 1.34 | **1.67** |
| 1377994_at | **Pmaip1** | Signaling | 159 | 1.04 | 1.37 | **1.60** |
| 1367957_at | **Rgs3** | Signaling | 1140 | 1.41 | 1.04 | **2.38** |
| 1392990_at | **Sox17** | Transcriptional regulation | 517 | 1.47 | 0.92 | **1.62** |
| 1384842_s_at | **Tnfrsf6** | Receptors | 456 | 1.02 | 1.18 | **1.55** |
| 1395645_at | **Unknown** | Unknown | 744 | 0.92 | 1.17 | **1.83** |
| 1381334_at | **Unknown** | Unknown | 219 | 0.91 | 1.02 | **1.74** |
|  |  |  |  |  |  |  |
| **PD184352/ET-1 downregulation of baseline RNA expression** | | |  |  |  |  |
| 1389230_at | **Arrdc3** | Not established | 2677 | 0.72 | 0.96 | **0.66** |
| 1371953_at | **Ccng2** | Signaling | 622 | 0.97 | 0.77 | **0.65** |
| 1379238_at | **Ctdspl** | Signaling | 256 | 0.71 | 0.69 | **0.66** |
| 1370097_a_at, 1373661_a_at, 1389244_x_at | **Cxcr4** | Receptors | 1642, 1564, 1585 | 0.71 | 0.79 | **0.58** |
| 1376084_a_at | **Espl1** | Protein synthesis/modification | 190 | 0.89 | 0.96 | **0.59** |
| 1380617_at | **Gimap5** | Signaling | 343 | 0.72 | 0.90 | **0.66** |
| 1383661_at | **Hs3st3b1** | Protein synthesis/modification | 100 | 0.67 | 0.91 | **0.60** |
| 1391320_at | **Intron:Hspbap1** | Introns | 134 | 1.11 | 0.85 | **0.67** |
| 1396973_at | **Intron:Phf15** | Introns | 108 | 1.11 | 0.81 | **0.53** |
| 1368486_at | **Irs3** | Signaling | 119 | 1.05 | 0.68 | **0.58** |
| 1379211_at | **Pdgfrb** | Receptors | 487 | 1.41 | 0.69 | **0.66** |
| 1379313_at | **Ptpre** | Receptors | 113 | 0.92 | 1.02 | **0.66** |
| 1389353_at | **Sema6d** | Receptors | 936 | 0.73 | 0.77 | **0.63** |
| 1388746_at | **Serpine3** | Protein synthesis/modification | 78 | 1.31 | 1.17 | **0.50** |
| 1389742_at | **Zfp217** | Transcriptional regulation | 530 | 0.76 | 0.83 | **0.59** |
| 1386721_at, 1393990_at | **Znf503** | Transcriptional regulation | 813, 905 | 0.70 | 0.80 | **0.53** |
