# Supplementary material for: ERK1/2 Signaling Dominates Over RhoA Signaling in Regulating Early Changes in RNA Expression Induced by Endothelin-1 in Neonatal Rat Cardiomyocytes
Source: PLoS One. 2010 Apr 2;5(4):e10027. doi: 10.1371/journal.pone.0010027 (PMC2848868; doi:10.1371/journal.pone.0010027)
Supplement: Table S5 — RNAs upregulated in cardiomyocytes by ET-1: effects of C3T. Cardiomyocytes were unstimulated (Control) or exposed to ET-1, C3T or ET-1 in the presence of C3T (C3T/ET-1). Microarray analysis was performed to identify RNAs significantly downregulated by ET-1 (>1.5-fold change, FDR<0.05) and with significant inhibition or enhancement with C3T. Raw values are provided for Controls and expression relative to Controls is provided for C3T, ET-1 and C3T/ET-1. Results are means for 4 separate hybridisations. Where multiple probesets represented the same RNA, individual raw values are provided for controls and, since the relative fold changes were similar, the mean values are provided for the treatments. RNAs in each group (inhibited by C3T, no significant effect of C3T) are listed alphabetically according to gene symbol. (0.23 MB DOC) [file pone.0010027.s005.doc]

**Table S5. RNAs downregulated in cardiomyocytes by ET-1: effects of C3T.** Cardiomyocytes were unstimulated (Control) or exposed to ET-1, C3T or ET-1 in the presence of C3T (C3T/ET-1). Microarray analysis was performed to identify RNAs significantly downregulated by ET-1 (>1.5-fold change, FDR<0.05) and with significant inhibition or enhancement with C3T. Raw values are provided for Controls and expression relative to Controls is provided for C3T, ET-1 and C3T/ET-1. Results are means for 4 separate hybridisations. Where multiple probesets represented the same RNA, individual raw values are provided for controls and, since the relative fold changes were similar, the mean values are provided for the treatments. RNAs in each group (inhibited by C3T, no significant effect of C3T) are listed alphabetically according to gene symbol.

| **Probeset** | **Gene symbol** | **Classification** | **Control** | **C3T** | **ET-1** | **C3T/ET-1** |
| --- | --- | --- | --- | --- | --- | --- |
|  |  |  | **(Raw values)** | **(Relative to controls)** | | |
| **Significant inhibition by C3T** | | |  |  |  |  |
| 1383848_at | **Adrb1** | Receptors | 627 | 1.19 | **0.44** | 0.61 |
| 1385627_at, 1398482_at | **Bcl3** | Transcription | 652, 503 | 1.55 | **0.48** | 0.73 |
| 1368813_at, 1387343_at | **Cebpd** | Transcription | 1479, 2974 | 1.10 | **0.42** | 0.70 |
| 1390024_at | **Clec2d/g** | Receptors | 1686 | 1.05 | **0.64** | 0.99 |
| 1383205_at | **Dact2** | Not established | 186 | 1.00 | **0.66** | 1.30 |
| 1387036_at | **Hes1** | Transcription | 1576 | 1.29 | **0.58** | 0.87 |
| 1387028_a_at | **Id1** | Transcription | 3595 | 1.20 | **0.61** | 0.91 |
| 1392578_at | **Intron:Mgp** | Introns | 626 | 1.05 | **0.21** | 0.65 |
| 1393406_at | **Intron:Rbx1** | Introns | 311 | 1.10 | **0.65** | 0.89 |
| 1368073_at | **Irf1** | Transcription | 907 | 1.06 | **0.41** | 0.57 |
| 1368249_at, 1381396_s_at | **Klf15** | Transcription | 931, 288 | 0.94 | **0.40** | 0.34 |
| 1381267_at | **LOC691468** | Not established | 138 | 1.08 | **0.57** | 0.81 |
| 1388525_at | **Pik3ip1** | Signaling | 1024 | 0.86 | **0.53** | 0.44 |
| 1378484_at | **Rasl12** | Signaling | 684 | 0.62 | **0.58** | 0.38 |
| 1374364_at | **RGD1562335** | Not established | 392 | 1.06 | **0.51** | 0.68 |
| 1374756_at | **RGD1562665** | Not established | 181 | 1.15 | **0.35** | 0.75 |
| 1393262_at | **Smad9** | Transcription | 454 | 0.95 | **0.65** | 0.56 |
| 1373219_at | **Snai1** | Transcription | 1379 | 1.04 | **0.27** | 0.34 |
| 1397220_at | **Snhg7** | Non-protein-coding | 657 | 1.22 | **0.64** | 0.87 |
| 1369584_at, 1377092_at | **Socs3** | Signaling | 732, 2404 | 1.29 | **0.56** | 0.84 |
| 1390215_at | **Ston2** | Signaling | 127 | 0.83 | **0.64** | 0.99 |
| 1371131_a_at | **Txnip** | Transcription | 5283 | 1.16 | **0.65** | 0.87 |
| 1389084_at | **Unknown** | Unknown | 377 | 0.98 | **0.47** | 0.59 |
| 1380914_at, 1393652_at | **Zbtb1** | Transcription | 209, 534 | 1.09 | **0.64** | 0.58 |
| 1386633_at | **Zfp869** | Transcription | 276 | 1.04 | **0.60** | 0.77 |
| 1391507_at | **Znf467** | Transcription | 385 | 0.70 | **0.60** | 0.42 |
|  |  |  |  |  |  |  |
| **No significant effect of C3T** | | |  |  |  |  |
| 1390931_at | **Adamts15** | Protein synthesis/modification | 1070 | 1.13 | **0.54** | 0.64 |
| 1390974_a_at | **Adat2** | RNA binding/processing | 392 | 0.87 | **0.61** | 0.75 |
| 1381153_at | **Anapc4** | Protein synthesis/modification | 284 | 0.86 | **0.58** | 0.66 |
| 1389264_at | **Ankrd54** | Not established | 376 | 0.90 | **0.57** | 0.62 |
| 1373287_at | **Atoh8** | Transcription | 863 | 1.05 | **0.43** | 0.45 |
| 1370823_at | **Bambi** | Receptors | 960 | 0.86 | **0.62** | 0.62 |
| 1374493_at | **Bmf** | Signaling | 424 | 0.81 | **0.63** | 0.62 |
| 1384214_a_at | **Ccnt2** | Transcription | 211 | 1.07 | **0.54** | 0.62 |
| 1389145_at | **Cdc42ep2** | Signaling | 635 | 1.52 | **0.61** | 0.75 |
| 1398710_at | **Cyp2u1** | Metabolism | 427 | 0.97 | **0.58** | 0.49 |
| 1368025_at | **Ddit4** | Signaling | 1910 | 0.87 | **0.30** | 0.33 |
| 1376612_at | **Dtwd1** | Not established | 252 | 0.97 | **0.65** | 0.60 |
| 1373114_at | **Dtx4** | Signaling | 1566 | 0.87 | **0.65** | 0.60 |
| 1393058_at | **Eid2** | Transcription | 490 | 1.15 | **0.62** | 0.74 |
| 1391741_a_at | **Fam78a** | Not established | 338 | 0.98 | **0.63** | 0.52 |
| 1390995_at | **Fkbpl** | Protein synthesis/modification | 316 | 0.91 | **0.65** | 0.64 |
| 1389404_at | **Foxs1** | Transcription | 553 | 1.02 | **0.54** | 0.64 |
| 1374530_at | **Fzd7** | Receptors | 339 | 0.86 | **0.66** | 0.60 |
| 1389871_at | **Got2** | Metabolism | 91 | 1.03 | **0.63** | 0.61 |
| 1374234_at | **Hkr3** | Transcription | 246 | 1.14 | **0.66** | 0.82 |
| 1389675_at, 1391026_at | **Ier5l** | Not established | 999, 1430 | 0.89 | **0.32** | 0.34 |
| 1378945_at | **Intron:Aco2** | Introns | 241 | 0.92 | **0.58** | 0.68 |
| 1396420_at | **Intron:Baz1b** | Introns | 170 | 1.21 | **0.64** | 0.74 |
| 1385506_at | **Intron:Cdon** | Introns | 298 | 1.00 | **0.58** | 0.50 |
| 1392650_at | **Intron:Csnk2a1** | Introns | 320 | 1.05 | **0.63** | 0.73 |
| 1398560_at | **Intron:Dscr1l1** | Introns | 145 | 0.63 | **0.41** | 0.28 |
| 1380168_at | **Intron:Etv4** | Introns | 140 | 1.00 | **0.66** | 0.72 |
| 1376522_at | **Intron:Fabp3** | Introns | 318 | 0.91 | **0.46** | 0.44 |
| 1391171_at | **Intron:Itm2b** | Introns | 303 | 0.99 | **0.59** | 0.58 |
| 1393540_at | **Intron:Myh6** | Introns | 512 | 0.83 | **0.62** | 0.56 |
| 1392317_at | **Intron:Scl33a1** | Introns | 121 | 0.99 | **0.49** | 0.60 |
| 1397165_at | **Intron:Smarca2** | Introns | 148 | 0.90 | **0.62** | 0.69 |
| 1374786_at | **Intron:Tia1** | Introns | 151 | 1.17 | **0.49** | 0.60 |
| 1390776_at, 1394251_x_at | **Irx3** | Transcription | 1232, 566 | 1.36 | **0.66** | 0.87 |
| 1390969_at | **Kcne4** | Channels/pumps/transporters | 1275 | 0.84 | **0.62** | 0.60 |
| 1389988_at | **Kctd2** | Channels/pumps/transporters | 989 | 1.07 | **0.67** | 0.68 |
| 1378332_at, 1389479_at, 1393150_at | **Klf3** | Transcription | 1254, 1744, 1242 | 0.93 | **0.60** | 0.60 |
| 1381065_at | **LOC499120** | Not established | 670 | 1.04 | **0.64** | 0.73 |
| 1385431_at | **LOC688018** | Signaling | 250 | 0.69 | **0.63** | 0.65 |
| 1372060_at | **Lysmd4** | Not established | 413 | 0.87 | **0.56** | 0.50 |
| 1376718_at | **Mblac1** | Not established | 499 | 0.95 | **0.61** | 0.67 |
| 1396481_at | **Mirn1-2/-133a-1** | Non-protein coding | 75 | 0.77 | **0.34** | 0.25 |
| 1374693_at | **Parp16** | Metabolism | 459 | 0.82 | **0.55** | 0.50 |
| 1384262_at | **Ppp1r3b** | Signaling | 487 | 0.86 | **0.49** | 0.51 |
| 1384937_at | **Rbak** | Transcription | 112 | 1.04 | **0.53** | 0.53 |
| 1385253_at | **RGD1304610** | Not established | 173 | 0.95 | **0.64** | 0.61 |
| 1380314_at | **RGD1563072** | Not established | 280 | 0.85 | **0.66** | 0.67 |
| 1392170_at | **Rwdd3** | Not established | 180 | 1.10 | **0.66** | 0.65 |
| 1367920_at | **S1pr2** | Receptors | 622 | 0.94 | **0.61** | 0.75 |
| 1397522_at | **Sbf1** | Signaling | 315 | 0.79 | **0.65** | 0.78 |
| 1390249_at | **Sept14** | Signaling | 503 | 1.09 | **0.65** | 0.67 |
| 1375121_at, 1394025_at | **Smad6** | Transcription | 944, 650 | 0.94 | **0.63** | 0.66 |
| 1383589_at | **Snai2** | Transcription | 515 | 0.84 | **0.62** | 0.50 |
| 1372633_at | **Spg20** | Signaling | 684 | 0.88 | **0.64** | 0.61 |
| 1388786_at | **Synpo** | Cytoskeleton/myofibrillar | 2163 | 0.90 | **0.66** | 0.62 |
| 1393153_at | **Tardbp** | RNA binding/processing | 234 | 0.89 | **0.66** | 0.61 |
| 1383282_at | **Thap11** | DNA regulation | 566 | 1.08 | **0.65** | 0.75 |
| 1397692_at | **Tia1** | Protein synthesis/modification | 133 | 0.98 | **0.52** | 0.63 |
| 1390928_at | **Tigd2** | DNA regulation | 267 | 0.75 | **0.41** | 0.34 |
| 1393649_at | **Tmpo** | Transcription | 102 | 1.08 | **0.55** | 0.73 |
| 1397527_at | **Trim47** | Not established | 784 | 0.88 | **0.54** | 0.65 |
| 1391027_at | **Trim65** | Not established | 370 | 0.91 | **0.58** | 0.61 |
| 1376358_at | **Unknown** | Unknown | 98 | 1.12 | **0.45** | 0.70 |
| 1377045_at | **Unknown** | Unknown | 286 | 0.98 | **0.58** | 0.67 |
| 1377629_at | **Unknown** | Unknown | 380 | 0.90 | **0.65** | 0.59 |
| 1378117_at | **Unknown** | Unknown | 317 | 0.96 | **0.64** | 0.55 |
| 1378367_at | **Unknown** | Unknown | 143 | 1.18 | **0.61** | 0.52 |
| 1378547_at | **Unknown** | Unknown | 437 | 0.93 | **0.66** | 0.81 |
| 1379454_at | **Unknown** | Unknown | 150 | 0.90 | **0.65** | 0.81 |
| 1381077_at | **Unknown** | Unknown | 176 | 0.91 | **0.63** | 0.70 |
| 1381619_at | **Unknown** | Unknown | 131 | 1.27 | **0.38** | 0.47 |
| 1383058_at | **Unknown** | Unknown | 2498 | 0.91 | **0.45** | 0.48 |
| 1383919_at | **Unknown** | Unknown | 154 | 0.87 | **0.64** | 0.63 |
| 1383977_a_at | **Unknown** | Unknown | 152 | 1.06 | **0.37** | 0.53 |
| 1389419_at | **Unknown** | Unknown | 576 | 0.98 | **0.55** | 0.58 |
| 1389714_at | **Unknown** | Unknown | 201 | 0.79 | **0.65** | 0.77 |
| 1391863_at | **Unknown** | Unknown | 348 | 1.16 | **0.18** | 0.24 |
| 1392605_at | **Unknown** | Unknown | 181 | 0.87 | **0.62** | 0.72 |
| 1393512_at | **Unknown** | Unknown | 192 | 1.00 | **0.63** | 0.56 |
| 1377154_at | **Zfp157** | Transcription | 251 | 0.86 | **0.58** | 0.50 |
| 1372205_at | **Zfp278** | Transcription | 417 | 0.99 | **0.66** | 0.63 |
| 1393438_at | **Zfp316** | Transcription | 203 | 0.94 | **0.62** | 0.75 |
| 1370984_at | **Zfp46** | Transcription | 660 | 0.92 | **0.66** | 0.61 |
| 1386793_at | **Zfp61** | Transcription | 183 | 0.91 | **0.42** | 0.40 |
| 1381282_at | **Zfp768** | Transcription | 277 | 0.62 | **0.64** | 0.51 |
| 1385597_at | **Znf124** | Transcription | 83 | 0.88 | **0.39** | 0.28 |
| 1389366_at | **Znf553** | Transcription | 432 | 0.90 | **0.51** | 0.60 |
| 1372699_at | **Znf775** | Transcription | 201 | 0.98 | **0.54** | 0.54 |
| 1384432_at | **Zscan12** | Transcription | 199 | 1.05 | **0.62** | 0.59 |
