# Supplementary material for: ERK1/2 Signaling Dominates Over RhoA Signaling in Regulating Early Changes in RNA Expression Induced by Endothelin-1 in Neonatal Rat Cardiomyocytes
Source: PLoS One. 2010 Apr 2;5(4):e10027. doi: 10.1371/journal.pone.0010027 (PMC2848868; doi:10.1371/journal.pone.0010027)
Supplement: Table S6 — RNAs regulated in cardiomyocytes by C3T alone or C3T in the presence of ET-1. Cardiomyocytes were unstimulated (Control) or exposed to ET-1, C3T or ET-1 in the presence of C3T (C3T/ET-1). Microarray analysis was performed. RNAs not significantly regulated by ET-1 alone but significantly regulated by C3T or C3T/ET-1 were selected (>1.5-fold change, FDR<0.05) and clustered according to upregulation or downregulation. Raw values are provided for Controls and expression relative to Controls is provided for C3T, ET-1 and C3T/ET-1. Results are means for 4 separate hybridisations. Where multiple probesets represented the same RNA, individual raw values are provided for controls and, since the relative fold changes were similar, the mean values are provided for the treatments. RNAs in each group are listed alphabetically according to gene symbol. (0.84 MB DOC) [file pone.0010027.s006.doc]

**Table S6. RNAs regulated in cardiomyocytes by C3T alone or C3T in the presence of ET-1.** Cardiomyocytes were unstimulated (Control) or exposed to ET-1, C3T or ET-1 in the presence of C3T (C3T/ET-1). Microarray analysis was performed. RNAs not significantly regulated by ET-1 alone but significantly regulated by C3T or C3T/ET-1 were selected (>1.5-fold change, FDR<0.05) and clustered according to upregulation or downregulation. Raw values are provided for Controls and expression relative to Controls is provided for C3T, ET-1 and C3T/ET-1. Results are means for 4 separate hybridisations. Where multiple probesets represented the same RNA, individual raw values are provided for controls and, since the relative fold changes were similar, the mean values are provided for the treatments. RNAs in each group are listed alphabetically according to gene symbol.

| **Probeset** | **Gene symbol** | **Classification** | **Control** | **C3T** | **ET-1** | **C3T/ET-1** |
| --- | --- | --- | --- | --- | --- | --- |
|  |  |  | **(Raw values)** | **(Relative to controls)** | | |
| **C3T upregulation of baseline RNA expression** | | |  |  |  |  |
| 1388924_at | **Angptl4** | Agonists | 657 | **2.55** | 0.75 | 1.84 |
| 1379483_at | **AS:Bhlhe40** | Potential AS | 925 | **2.01** | 1.31 | 2.09 |
| 1393252_at | **AS:Fbln1** | Potential AS | 6380 | **1.54** | 1.14 | 1.43 |
| 1393006_at | **AS:Fubp3** | Potential AS | 120 | **1.62** | 1.25 | 1.44 |
| 1381990_at | **AS:Galnt1** | Potential AS | 137 | **1.88** | 1.15 | 1.79 |
| 1389905_at | **AS:Gas1** | Potential AS | 2452 | **1.65** | 0.92 | 1.21 |
| 1377413_at | **AS:Lsm14a** | Potential AS | 193 | **1.53** | 1.22 | 1.57 |
| 1376848_at | **AS:Myo10** | Potential AS | 1113 | **1.66** | 0.96 | 1.70 |
| 1382365_at | **AS:Nlk** | Potential AS | 232 | **1.57** | 0.96 | 1.45 |
| 1393494_at | **AS:Pdgf1** | Potential AS | 367 | **1.65** | 0.97 | 1.80 |
| 1375687_at | **AS:Rab14** | Potential AS | 3363 | **1.51** | 1.09 | 1.35 |
| 1383599_at | **AS:Rbbp4** | Potential AS | 4261 | **1.52** | 1.12 | 1.42 |
| 1375921_at | **AS:Rcn1** | Potential AS | 985 | **1.64** | 1.17 | 1.63 |
| 1375268_at | **AS:Rps27l** | Potential AS | 1028 | **1.53** | 1.30 | 1.54 |
| 1380200_at | **AS:Zeb1** | Potential AS | 118 | **1.68** | 1.07 | 1.36 |
| 1374117_at | **Baiap2** | Signaling | 150 | **1.62** | 1.15 | 1.58 |
| 1385853_at | **Baz1a** | Transcription | 131 | **1.54** | 1.10 | 1.63 |
| 1374034_at | **Cars** | Protein synthesis/modification | 882 | **1.67** | 1.17 | 1.91 |
| 1369814_at | **Ccl20** | Agonists | 340 | **1.52** | 1.10 | 1.77 |
| 1368921_a_at, 1387952_a_at, 1390659_at | **Cd44** | Adhesion/ECM | 852, 1236, 1772 | **1.69** | 1.32 | 2.21 |
| 1379381_at | **Ciita** | Transcription | 78 | **1.50** | 1.40 | 1.34 |
| 1384969_at | **Col24a1** | Adhesion/ECM | 274 | **1.57** | 1.01 | 1.49 |
| 1387170_at | **Csnk2a1** | Signaling | 268 | **1.77** | 0.91 | 1.43 |
| 1368200_at | **Cx3cl1** | Agonists | 869 | **1.57** | 0.92 | 1.33 |
| 1387316_at | **Cxcl1** | Agonists | 6292 | **2.16** | 1.40 | 2.26 |
| 1368760_at | **Cxcl2** | Agonists | 576 | **1.74** | 1.31 | 3.00 |
| 1370633_at, 1370634_x_at, 1388032_a_at | **Cxcl3** | Agonists | 929, 936, 772 | **1.56** | 1.15 | 2.08 |
| 1387648_at | **Cxcl5** | Agonists | 2152 | **2.22** | 0.98 | 2.16 |
| 1397508_at | **Ddx18** | RNA binding/processing | 185 | **1.51** | 0.90 | 1.41 |
| 1389803_at | **Erbb4** | Receptors | 103 | **1.56** | 1.33 | 1.29 |
| 1379077_at | **Fam181b** | Not established | 76 | **1.64** | 0.94 | 0.84 |
| 1374643_at | **Fat4** | Adhesion/ECM | 234 | **1.52** | 1.22 | 1.55 |
| 1372016_at | **Gadd45b** | Signaling | 618 | **1.64** | 1.37 | 1.85 |
| 1387659_at | **Gda** | Metabolism | 2724 | **1.54** | 1.06 | 1.45 |
| 1380410_at, 1385926_at | **Glipr2** | Not established | 483, 358 | **1.64** | 1.21 | 1.62 |
| 1379535_at | **Intron:Ccnh** | Introns | 78 | **1.52** | 1.20 | 1.22 |
| 1397045_at | **Intron:Chst11** | Introns | 99 | **1.66** | 1.28 | 1.51 |
| 1378843_at | **Intron:Dnajb6** | Introns | 102 | **1.57** | 1.48 | 1.53 |
| 1378783_at, 1379114_at, 1396378_at | **Intron:Mast4** | Introns | 351, 120, 214 | **1.78** | 1.18 | 1.62 |
| 1376098_a_at | **Intron:Myo1g** | Introns | 758 | **1.55** | 1.49 | 1.91 |
| 1381133_at | **Intron:Plcb1** | Introns | 112 | **1.55** | 0.99 | 1.33 |
| 1385177_at | **Intron:Sorbs1** | Introns | 100 | **1.54** | 1.50 | 1.36 |
| 1395372_at | **Itgb8** | Adhesion/ECM | 217 | **1.67** | 0.96 | 1.03 |
| 1395966_at | **Kctd14** | Channels/pumps/transporters | 145 | **1.52** | 0.80 | 1.11 |
| 1369610_at | **Lin7c** | Trafficking | 221 | **1.53** | 1.32 | 1.30 |
| 1390790_a_at | **Lipg** | Metabolism | 154 | **1.56** | 1.04 | 1.78 |
| 1374594_at, 1397729_x_at | **LOC363060** | Not established | 54, 67 | **7.10** | 2.12 | 7.92 |
| 1374028_at | **LOC500974** | Not established | 263 | **1.55** | 1.07 | 1.38 |
| 1369393_at | **Map3k8** | Signaling | 219 | **1.58** | 0.82 | 1.32 |
| 1369410_at | **Mitochondrial genome** | DNA regulation | 871 | **1.84** | 1.39 | 1.74 |
| 1368657_at | **Mmp3** | Protein synthesis/modification | 112 | **1.57** | 1.18 | 1.98 |
| 1370968_at | **Nfkb1** | Transcription | 2247 | **1.51** | 1.00 | 1.79 |
| 1389538_at | **Nfkbia** | Transcription | 1524 | **1.96** | 0.71 | 1.42 |
| 1368438_at, 1370669_a_at, 1379607_at, 1385381_at | **Pde10a** | Signaling | 550, 415, 389, 347 | **2.12** | 0.94 | 1.62 |
| 1376737_at | **Plcxd3** | Signaling | 1049 | **1.58** | 0.99 | 1.53 |
| 1379312_at | **Pprc1** | Transcription | 491 | **1.52** | 1.06 | 1.53 |
| 1368014_at | **Ptges** | Metabolism | 271 | **1.86** | 1.16 | 2.15 |
| 1373923_at, 1384391_at, 1393351_at | **Rdh10** | Metabolism | 340, 244, 337 | **1.82** | 0.99 | 1.85 |
| 1382489_at | **RGD1564964** | Not established | 325 | **1.55** | 1.18 | 1.01 |
| 1373777_at | **Rgs16** | Signaling | 547 | **1.50** | 1.39 | 1.81 |
| 1367849_at, 1376062_at | **Sdc1** | Adhesion/ECM | 1447, 195 | **1.55** | 1.31 | 2.09 |
| 1392556_at | **Shroom3** | Cytoskeleton/myofibrillar | 262 | **1.50** | 1.06 | 1.31 |
| 1374845_at | **Slc25a37** | Channels/pumps/transporters | 353 | **1.59** | 0.93 | 1.49 |
| 1377916_at | **Slfn2** | Not established | 1195 | **1.82** | 1.16 | 2.11 |
| 1367998_at | **Slpi** | Protein synthesis/modification | 3863 | **2.14** | 0.99 | 1.81 |
| 1387617_at | **Tpm3** | Cytoskeleton/myofibrillar | 757 | **1.80** | 1.30 | 1.79 |
| 1392598_at | **Unknown** | Unknown | 1153 | **1.80** | 1.21 | 1.93 |
| 1377823_at | **Unknown** | Unknown | 311 | **1.80** | 0.98 | 1.55 |
| 1398703_at | **Unknown** | Unknown | 210 | **1.70** | 1.13 | 1.42 |
| 1385569_at | **Unknown** | Unknown | 250 | **1.68** | 0.98 | 1.61 |
| 1397316_at | **Unknown** | Unknown | 107 | **1.57** | 1.18 | 1.06 |
| 1384562_at | **Unknown** | Unknown | 538 | **1.56** | 1.31 | 1.90 |
| 1377889_at | **Urb1** | Protein synthesis/modification | 209 | **1.52** | 1.11 | 1.50 |
| 1393140_at | **Zc3h12a** | Transcription | 347 | **1.64** | 1.17 | 1.38 |
|  |  |  |  |  |  |  |
| **C3T downregulation of baseline RNA expression** | | |  |  |  |  |
| 1382296_at, 1394483_at | **Adamts5** | Protein synthesis/modification | 299, 1052 | **0.63** | 0.98 | 0.60 |
| 1371824_at | **Ak3l1** | Metabolism | 414 | **0.64** | 0.89 | 0.64 |
| 1387796_at | **Alox15** | Metabolism | 497 | **0.67** | 0.88 | 0.66 |
| 1388370_at, 1392937_at | **Ccni** | Signaling | 4950, 1765 | **0.61** | 0.90 | 0.57 |
| 1369083_at, 1382809_at | **Cirbp** | RNA binding/processing | 457, 361 | **0.57** | 0.82 | 0.58 |
| 1368648_at | **Cox4i2** | Metabolism | 584 | **0.51** | 0.91 | 0.50 |
| 1367739_at | **Cox8h** | Metabolism | 3838 | **0.47** | 1.06 | 0.48 |
| 1387874_at | **Dbp** | Transcription | 504 | **0.66** | 0.82 | 0.52 |
| 1368174_at | **Egln3** | Metabolism | 3489 | **0.62** | 1.12 | 0.75 |
| 1372735_at | **Eif3k** | Protein synthesis/modification | 3241 | **0.62** | 0.94 | 0.55 |
| 1389297_at | **Ero1l** | Protein synthesis/modification | 1903 | **0.61** | 1.08 | 0.67 |
| 1377713_at | **Foxn3** | Transcription | 1493 | **0.61** | 0.94 | 0.62 |
| 1379654_at | **Giyd2** | DNA regulation | 231 | **0.60** | 0.78 | 0.52 |
| 1379939_a_at | **Intron:RGD1306063** | Introns | 337 | **0.66** | 0.95 | 0.62 |
| 1374060_at | **Irs1** | Signaling | 940 | **0.65** | 0.79 | 0.55 |
| 1393310_at | **Itch** | Protein synthesis/modification | 529 | **0.63** | 0.92 | 0.69 |
| 1393226_at | **Itgav** | Adhesion/ECM | 1208 | **0.61** | 1.11 | 0.73 |
| 1372737_at | **Ldb3** | Cytoskeleton/myofibrillar | 1430 | **0.66** | 0.95 | 0.77 |
| 1367628_at | **Lgals1** | Adhesion/ECM | 7415 | **0.62** | 0.99 | 0.63 |
| 1373387_at | **LOC688717** | Not established | 2402 | **0.65** | 1.02 | 0.64 |
| 1385487_at | **Lsm1** | RNA binding/processing | 952 | **0.64** | 1.04 | 0.83 |
| 1367609_at | **Mif** | Agonists | 7209 | **0.64** | 0.98 | 0.62 |
| 1377720_x_at | **Mitochondrial genome** | DNA regulation | 6917 | **0.67** | 0.98 | 0.66 |
| 1372623_at | **Mlec** | Metabolism | 660 | **0.66** | 1.06 | 0.95 |
| 1380447_a_at | **Mrps18c** | Protein synthesis/modification | 796 | **0.60** | 1.02 | 0.58 |
| 1372295_at, 1384009_at | **Narf** | Cytoskeleton/myofibrillar | 1136, 456 | **0.59** | 0.85 | 0.54 |
| 1375220_at | **Ndufa11** | Metabolism | 7788 | **0.65** | 0.99 | 0.60 |
| 1388489_at | **Ndufa3** | Metabolism | 4953 | **0.55** | 0.94 | 0.54 |
| 1386951_at | **Ndufa5** | Metabolism | 3773 | **0.50** | 0.96 | 0.50 |
| 1389059_at | **Nfix** | Transcription | 1638 | **0.66** | 0.97 | 0.60 |
| 1367479_at | **Nola3** | RNA binding/processing | 4989 | **0.61** | 1.01 | 0.61 |
| 1371166_at | **Nos3** | Metabolism | 494 | **0.64** | 0.95 | 0.60 |
| 1381888_x_at | **Pacrgl** | Not established | 98 | **0.57** | 0.88 | 0.88 |
| 1384824_at | **Pcdh18** | Adhesion/ECM | 590 | **0.64** | 0.82 | 0.69 |
| 1398884_at | **Pfdn5** | Protein synthesis/modification | 3092 | **0.62** | 0.97 | 0.62 |
| 1383749_at | **Phospho1** | Metabolism | 344 | **0.51** | 0.88 | 0.58 |
| 1375053_at | **Pknox2** | Transcription | 375 | **0.66** | 0.99 | 0.76 |
| 1372556_at | **Polr2l** | Transcription | 4363 | **0.60** | 0.99 | 0.58 |
| 1374884_at | **Ppm1d** | Signaling | 206 | **0.52** | 0.79 | 0.70 |
| 1367813_at | **Ppp1r14a** | Signaling | 415 | **0.53** | 0.84 | 0.63 |
| 1393638_at | **Ptger4** | Receptors | 632 | **0.51** | 1.05 | 0.63 |
| 1370982_at, 1375476_at | **Pygm** | Metabolism | 2319, 1510 | **0.65** | 0.83 | 0.68 |
| 1389033_at | **RGD1306917** | Not established | 2172 | **0.59** | 0.96 | 0.58 |
| 1373596_at | **RGD1310423** | Not established | 659 | **0.60** | 0.71 | 0.58 |
| 1383643_at | **RGD1560328** | Not established | 6011 | **0.61** | 0.96 | 0.61 |
| 1371410_at | **RGD1564058** | Not established | 3824 | **0.64** | 0.98 | 0.64 |
| 1377728_at | **RGD1565641** | Not established | 1848 | **0.61** | 0.99 | 0.58 |
| 1372585_at | **RGD1566254** | Not established | 1641 | **0.66** | 0.83 | 0.64 |
| 1371573_at | **Rpl36a** | Protein synthesis/modification | 13224 | **0.58** | 1.09 | 0.59 |
| 1376110_at | **Rpp25** | RNA binding/processing | 1513 | **0.59** | 0.70 | 0.44 |
| 1375954_at | **S100a13** | Channels/pumps/transporters | 760 | **0.54** | 0.85 | 0.58 |
| 1391387_s_at | **Slbp** | RNA binding/processing | 523 | **0.65** | 0.88 | 0.61 |
| 1368965_at | **Slc16a3** | Channels/pumps/transporters | 3321 | **0.65** | 0.96 | 0.65 |
| 1387130_at | **Slc40a1** | Channels/pumps/transporters | 725 | **0.64** | 0.84 | 0.56 |
| 1368082_at | **Slc4a2** | Channels/pumps/transporters | 725 | **0.64** | 0.93 | 0.57 |
| 1397584_at | **Snora78** | Non-protein-coding | 647 | **0.42** | 0.85 | 0.42 |
| 1387138_at | **Tac2** | Agonists | 496 | **0.66** | 0.85 | 0.62 |
| 1388782_at | **Tcf21** | Transcription | 1657 | **0.65** | 1.28 | 0.78 |
| 1390832_at | **Tmcc3** | Not established | 852 | **0.64** | 0.69 | 0.37 |
| 1371452_at | **Ubl7** | Protein synthesis/modification | 1177 | **0.58** | 0.88 | 0.57 |
| 1392935_at | **Unknown** | Unknown | 950 | **0.65** | 0.98 | 0.70 |
| 1388754_at | **Unknown** | Unknown | 3212 | **0.59** | 0.88 | 0.60 |
| 1382280_at | **Uqcrc2** | Metabolism | 3858 | **0.59** | 0.80 | 0.59 |
| 1397604_at | **Vbp1** | Protein synthesis/modification | 355 | **0.45** | 0.80 | 0.47 |
| 1368067_at | **Zfp148** | Transcription | 1128 | **0.60** | 0.88 | 0.53 |
|  |  |  |  |  |  |  |
| **C3T/ET-1 upregulation of baseline RNA expression** | | |  |  |  |  |
| 1387101_at | **Acsl4** | Metabolism | 2518 | 1.17 | 1.36 | **1.52** |
| 1393730_at | **Adamts4** | Protein synthesis/modification | 790 | 1.31 | 1.11 | **1.66** |
| 1390383_at | **Adfp** | Metabolism | 4251 | 1.17 | 1.41 | **1.74** |
| 1368946_at, 1388813_at | **Arf2** | Signaling | 597, 715 | 1.21 | 1.34 | **1.57** |
| 1375014_at | **Arl4c** | Signaling | 371 | 1.28 | 1.18 | **1.50** |
| 1386521_at | **AS:Akap2** | Potential AS | 129 | 1.09 | 1.23 | **1.56** |
| 1375183_at, 1385923_at | **AS:Id4** | Potential AS | 198, 97 | 1.23 | 1.58 | **1.81** |
| 1390983_at | **AS:Itga6** | Potential AS | 144 | 1.31 | 1.29 | **1.78** |
| 1375676_at | **AS:Lin7c** | Potential AS | 342 | 1.39 | 1.16 | **1.73** |
| 1391424_at | **AS:MiRNA cluster** | Potential AS | 116 | 1.04 | 1.27 | **1.64** |
| 1371130_at | **AS:Mmd** | Potential AS | 150 | 1.23 | 1.15 | **1.81** |
| 1379747_at | **AS:Prss35** | Potential AS | 355 | 1.38 | 1.21 | **2.20** |
| 1375986_at | **AS:Ptgs2** | Potential AS | 194 | 1.36 | 1.46 | **2.34** |
| 1394643_at | **AS:Qtrtd1** | Potential AS | 310 | 1.44 | 1.10 | **1.52** |
| 1385706_at | **AS:Tes** | Potential AS | 77 | 1.20 | 1.41 | **2.15** |
| 1371193_at | **AS:Tnfaip6** | Potential AS | 41 | 2.80 | 3.27 | **6.16** |
| 1371980_at | **Atad3a** | DNA regulation | 577 | 1.30 | 0.92 | **1.51** |
| 1367624_at | **Atf4** | Transcription | 7702 | 1.16 | 1.39 | **1.53** |
| 1389397_at | **Atp13a3** | Channels/pumps/transporters | 842 | 1.23 | 1.18 | **1.57** |
| 1370050_at, 1386426_at | **Atp2b1** | Channels/pumps/transporters | 1321, 1005 | 1.42 | 1.10 | **1.69** |
| 1378134_at | **Atp8b1** | Channels/pumps/transporters | 435 | 1.19 | 1.45 | **1.83** |
| 1385254_at | **Atr** | Signaling | 80 | 1.22 | 1.06 | **1.55** |
| 1370869_at | **Bcat1** | Metabolism | 1460 | 1.17 | 1.06 | **1.58** |
| 1368118_at | **Bcl10** | Signaling | 1596 | 1.22 | 1.30 | **1.52** |
| 1370650_s_at | **Bdkrb2** | Receptors | 119 | 1.67 | 1.21 | **2.98** |
| 1369815_at | **Ccl3** | Agonists | 939 | 1.25 | 0.97 | **1.78** |
| 1382603_at | **Cd274** | Receptors | 214 | 1.19 | 1.06 | **1.65** |
| 1387951_at | **Cd55** | Protein synthesis/modification | 160 | 1.11 | 1.22 | **1.92** |
| 1393365_at | **Chd1** | DNA regulation | 192 | 1.07 | 1.30 | **1.55** |
| 1393003_at | **Chst11** | Metabolism | 526 | 1.13 | 1.12 | **1.52** |
| 1386783_at | **ChSy-2** | Adhesion/ECM | 108 | 1.23 | 1.07 | **1.51** |
| 1370927_at | **Col12a1** | Adhesion/ECM | 917 | 1.22 | 1.24 | **1.54** |
| 1380287_at | **Creb5** | Transcription | 185 | 1.17 | 1.34 | **1.65** |
| 1368685_at | **Cspg4** | Adhesion/ECM | 257 | 1.53 | 1.04 | **1.82** |
| 1367940_at | **Cxcr7** | Receptors | 1294 | 1.37 | 1.30 | **1.60** |
| 1389155_at | **Dos** | Not established | 236 | 1.18 | 1.30 | **1.54** |
| 1388686_at | **Dscr1** | Signaling | 7390 | 1.09 | 1.42 | **1.54** |
| 1377023_at | **Dusp2** | Signaling | 454 | 1.09 | 1.38 | **1.68** |
| 1371840_at | **Edg1** | Receptors | 4385 | 0.97 | 1.45 | **1.54** |
| 1383353_at | **Efnb2** | Adhesion/ECM | 427 | 1.08 | 1.45 | **1.78** |
| 1369736_at, 1371527_at | **Emp1** | Signaling | 1574, 4157 | 0.86 | 1.41 | **1.55** |
| 1373535_at | **Enah** | Cytoskeleton/myofibrillar | 2302 | 0.97 | 1.26 | **1.52** |
| 1384687_at, 1388666_at | **Enc1** | Cytoskeleton/myofibrillar | 181, 1488 | 1.29 | 1.38 | **1.59** |
| 1377213_at | **Ern1** | Signaling | 354 | 1.17 | 1.38 | **1.76** |
| 1373917_at | **Etf1** | Protein synthesis/modification | 1789 | 1.19 | 1.30 | **1.52** |
| 1368851_at | **Ets1** | Transcription | 1805 | 1.01 | 1.43 | **1.51** |
| 1368550_at | **Foxq1** | Transcription | 152 | 1.17 | 1.15 | **1.62** |
| 1368947_at | **Gadd45a** | Signaling | 2344 | 1.08 | 1.44 | **1.76** |
| 1377761_at | **Gfpt2** | Metabolism | 4521 | 1.39 | 1.42 | **1.88** |
| 1367705_at, 1386908_at | **Glrx1** | Metabolism | 440, 581 | 1.00 | 1.36 | **1.60** |
| 1388953_at | **Gnl3** | Signaling | 1773 | 1.31 | 1.15 | **1.53** |
| 1388243_at | **Gpr176** | Receptors | 1384 | 1.10 | 1.29 | **1.50** |
| 1376828_at | **Gprc5a** | Receptors | 457 | 1.23 | 1.35 | **2.06** |
| 1370365_at | **Gss** | Metabolism | 575 | 1.21 | 1.10 | **1.68** |
| 1397587_at | **Hdac5** | Transcription | 175 | 1.11 | 1.37 | **1.57** |
| 1383685_at | **Heatr1** | RNA binding/processing | 310 | 1.34 | 1.22 | **1.59** |
| 1395012_at | **Herc4** | Protein synthesis/modification | 64 | 1.18 | 1.47 | **1.89** |
| 1387076_at | **Hif1a** | Transcription | 5540 | 1.41 | 1.19 | **1.67** |
| 1391560_at | **Hivep1** | Transcription | 317 | 1.14 | 1.26 | **1.68** |
| 1388309_at | **Hmga1** | Transcription | 2297 | 1.15 | 1.29 | **1.61** |
| 1368592_at, 1371170_a_at | **Il1a** | Agonists | 371, 283 | 1.15 | 0.98 | **1.66** |
| 1398256_at | **Il1b** | Agonists | 382 | 1.44 | 1.14 | **2.42** |
| 1392386_at | **Intron:Cflar** | Introns | 395 | 1.26 | 1.27 | **1.68** |
| 1374166_at | **Intron:Grtp1** | Introns | 1156 | 1.01 | 1.39 | **1.51** |
| 1397999_at | **Intron:Irs2** | Introns | 117 | 1.23 | 1.44 | **1.52** |
| 1377910_at | **Intron:Nol5** | Introns | 134 | 1.26 | 1.41 | **1.66** |
| 1382020_at | **Intron:Spag9** | Introns | 479 | 1.13 | 1.53 | **1.70** |
| 1390458_at | **Intron:Tnrc15** | Introns | 109 | 1.15 | 1.44 | **1.72** |
| 1384574_at | **Intron:Ugdh** | Introns | 147 | 1.44 | 1.34 | **1.71** |
| 1387346_at | **Itgb1** | Adhesion/ECM | 6243 | 1.29 | 1.10 | **1.51** |
| 1375866_at, 1390000_at | **Jmjd3** | Transcription | 570, 1124 | 1.20 | 1.41 | **1.79** |
| 1389374_at | **Kifc3** | Signaling | 446 | 1.11 | 0.93 | **1.50** |
| 1376089_at | **Ldlr** | Receptors | 3580 | 1.07 | 1.28 | **1.51** |
| 1373233_at, 1378179_a_at | **Lhfpl2** | Not established | 875, 788 | 1.19 | 1.23 | **1.60** |
| 1383906_at | **Lincr** | Protein synthesis/modification | 401 | 1.23 | 1.20 | **1.55** |
| 1368055_a_at | **Lmna** | Cytoskeleton/myofibrillar | 2888 | 1.09 | 1.30 | **1.57** |
| 1396949_at | **LOC298139** | Not established | 87 | 1.03 | 1.22 | **1.72** |
| 1378540_at | **LOC678970** | Not established | 651 | 1.32 | 1.14 | **1.55** |
| 1381434_s_at | **LOC681027** | Not established | 693 | 1.06 | 1.28 | **1.61** |
| 1376436_at | **Lysmd3** | Not established | 704 | 1.12 | 1.47 | **1.56** |
| 1388858_at | **Map2k3** | Signaling | 2308 | 1.18 | 1.37 | **1.95** |
| 1376690_at | **Med21** | Transcription | 763 | 1.08 | 1.36 | **1.54** |
| 1389911_at | **Metrnl** | Not established | 1972 | 1.06 | 1.46 | **1.64** |
| 1388990_at | **Mki67ip** | RNA binding/processing | 1445 | 1.28 | 1.22 | **1.53** |
| 1372808_at | **Mthfd2** | Metabolism | 805 | 1.28 | 1.34 | **1.78** |
| 1374797_at | **Ncapd2** | DNA regulation | 659 | 1.28 | 1.44 | **1.60** |
| 1375989_a_at | **Nfkb2** | Transcription | 849 | 1.43 | 1.14 | **1.76** |
| 1371259_at | **Ngfb** | Agonists | 227 | 1.41 | 1.16 | **2.24** |
| 1368173_at | **Nol5** | RNA binding/processing | 1937 | 1.29 | 1.33 | **1.52** |
| 1383614_at | **Nuak2** | Signaling | 319 | 1.28 | 1.49 | **1.97** |
| 1368747_at | **Nup98** | Signaling | 169 | 1.20 | 1.27 | **1.55** |
| 1388198_at | **Nupl1** | Signaling | 181 | 1.23 | 1.43 | **1.63** |
| 1374157_at | **Pde4b** | Signaling | 2177 | 1.21 | 1.17 | **1.63** |
| 1371684_at | **Pelo** | Protein synthesis/modification | 1834 | 1.20 | 1.43 | **1.73** |
| 1384950_at, 1395655_at | **Pi4k2b** | Signaling | 230, 800 | 1.26 | 1.30 | **1.61** |
| 1372725_at | **Plscr2** | Metabolism | 200 | 1.23 | 1.20 | **1.52** |
| 1392534_at | **Pmepa1** | Not established | 1691 | 1.18 | 1.14 | **1.51** |
| 1375061_at, 1396171_at | **Ppil4** | Protein synthesis/modification | 564, 496 | 1.12 | 1.34 | **1.54** |
| 1377858_at | **Prdm2** | Transcription | 120 | 1.28 | 1.45 | **2.14** |
| 1382154_at | **Ptpn12** | Signaling | 1974 | 1.23 | 1.23 | **1.65** |
| 1391899_at | **Purb** | Transcription | 188 | 1.17 | 1.59 | **1.70** |
| 1384089_at | **Rabgef1** | Signaling | 286 | 1.24 | 1.47 | **1.91** |
| 1390576_at | **Rbm15** | RNA binding/processing | 489 | 1.28 | 1.41 | **1.68** |
| 1393569_at | **Rbm18** | RNA binding/processing | 610 | 1.24 | 1.26 | **1.54** |
| 1382074_at | **Rnf19b** | Protein synthesis/modification | 437 | 1.31 | 1.25 | **1.75** |
| 1368914_at, 1395442_at | **Runx1** | Transcription | 545, 222 | 1.41 | 1.16 | **1.88** |
| 1386890_at | **S100a10** | Channels/pumps/transporters | 4808 | 1.09 | 1.38 | **1.62** |
| 1367721_at | **Sdc4** | Adhesion/ECM | 4388 | 1.46 | 1.38 | **1.97** |
| 1391946_at | **Selp** | Adhesion/ECM | 352 | 1.48 | 0.98 | **1.81** |
| 1399071_at | **Sertad2** | Transcription | 712 | 1.38 | 1.20 | **1.55** |
| 1388132_at | **Sfpq** | RNA binding/processing | 1352 | 1.22 | 1.26 | **1.52** |
| 1389203_at | **Sh3bp5l** | Not established | 495 | 1.31 | 1.29 | **1.87** |
| 1372896_at | **Similar to Hmm889** | Not established | 163 | 0.94 | 1.43 | **1.52** |
| 1369160_a_at, 1379739_at | **Slc4a7** | Channels/pumps/transporters | 175, 656 | 1.31 | 1.39 | **1.79** |
| 1368391_at, 1368392_at | **Slc7a1** | Channels/pumps/transporters | 1415, 1467 | 1.19 | 1.37 | **1.62** |
| 1390298_at | **Snag1** | Signaling | 1661 | 1.35 | 1.20 | **1.85** |
| 1383210_at | **Sox11** | Transcription | 240 | 1.12 | 1.23 | **1.51** |
| 1397618_at | **Spag9** | Signaling | 339 | 1.15 | 1.12 | **1.56** |
| 1377595_at | **Ssfa2** | Cytoskeleton/myofibrillar | 1358 | 1.08 | 1.43 | **1.57** |
| 1391871_at | **Stard13** | Signaling | 772 | 1.07 | 1.30 | **1.61** |
| 1371679_at | **Synpo2** | Cytoskeleton/myofibrillar | 3409 | 1.11 | 1.20 | **1.54** |
| 1383889_at | **Taf1a** | Transcription | 258 | 1.38 | 1.27 | **1.53** |
| 1371554_at | **Tcap** | Cytoskeleton/myofibrillar | 1402 | 1.22 | 1.46 | **1.95** |
| 1389409_at | **Tes** | Not established | 283 | 1.12 | 1.31 | **1.58** |
| 1383660_at | **Thoc4** | RNA binding/processing | 352 | 1.35 | 1.26 | **1.60** |
| 1367712_at | **Timp1** | Protein synthesis/modification | 8370 | 1.33 | 1.43 | **1.83** |
| 1379429_at | **Tmed5** | Signaling | 825 | 1.17 | 1.16 | **1.51** |
| 1374484_at | **Tmem39a** | Not established | 906 | 1.37 | 1.40 | **1.75** |
| 1384842_s_at | **Tnfrsf6** | Receptors | 456 | 1.35 | 1.18 | **1.73** |
| 1374945_at | **Trmt61a** | RNA binding/processing | 427 | 1.48 | 1.05 | **1.52** |
| 1370139_a_at | **Trpc6** | Channels/pumps/transporters | 74 | 1.20 | 0.98 | **1.76** |
| 1376100_at | **Tubb5/6** | Cytoskeleton/myofibrillar | 3533 | 1.04 | 1.29 | **1.54** |
| 1374893_at | **Uck2** | Metabolism | 794 | 1.14 | 1.09 | **1.58** |
| 1367938_at | **Ugdh** | Metabolism | 4151 | 1.37 | 1.44 | **1.94** |
| 1372442_at | **Uhmk1** | Signaling | 904 | 1.18 | 1.22 | **1.51** |
| 1380143_at | **Unknown** | Unknown | 386 | 1.65 | 1.12 | **1.87** |
| 1396251_at | **Unknown** | Unknown | 78 | 1.14 | 1.46 | **1.82** |
| 1386265_at | **Unknown** | Unknown | 209 | 1.18 | 1.27 | **1.80** |
| 1380365_at | **Unknown** | Unknown | 71 | 1.53 | 1.50 | **1.75** |
| 1395645_at | **Unknown** | Unknown | 744 | 1.36 | 1.17 | **1.67** |
| 1390778_at | **Unknown** | Unknown | 577 | 1.49 | 1.06 | **1.62** |
| 1391674_at | **Unknown** | Unknown | 289 | 1.28 | 1.18 | **1.61** |
| 1392184_at | **Unknown** | Unknown | 235 | 1.27 | 1.00 | **1.59** |
| 1390503_at | **Unknown** | Unknown | 199 | 1.10 | 1.42 | **1.55** |
| 1374940_at | **Usp36** | Protein synthesis/modification | 914 | 1.21 | 1.26 | **1.51** |
| 1388265_x_at | **Vcan** | Adhesion/ECM | 252 | 1.42 | 1.39 | **1.60** |
| 1383022_at, 1391217_at | **Zc3h12c** | Not established | 298, 447 | 1.35 | 1.27 | **1.56** |
| 1389152_at | **Zfyve27** | Not established | 251 | 1.53 | 1.20 | **1.69** |
|  |  |  |  |  |  |  |
| **C3T/ET-1 downregulation of baseline RNA expression** | | |  |  |  |  |
| 1373302_at, 1391791_at | **Acer2** | Metabolism | 374, 304 | 0.75 | 0.93 | **0.59** |
| 1377381_at | **Actn2** | Cytoskeleton/myofibrillar | 670 | 0.68 | 0.86 | **0.64** |
| 1395414_at | **Agl** | Metabolism | 190 | 0.85 | 0.71 | **0.66** |
| 1370708_a_at | **Akr1c14** | Metabolism | 329 | 0.64 | 0.64 | **0.55** |
| 1367985_at | **Alas2** | Metabolism | 322 | 0.83 | 0.87 | **0.64** |
| 1377725_at | **Anp32a** | Signaling | 599 | 0.68 | 0.82 | **0.66** |
| 1393051_at | **Armcx1** | Not established | 840 | 0.75 | 0.77 | **0.63** |
| 1375698_at | **AS:Rpl7** | Potential AS | 529 | 0.95 | 0.73 | **0.62** |
| 1381838_at | **Bivm** | Not established | 329 | 1.01 | 0.79 | **0.65** |
| 1367898_at | **Bnip3l** | Signaling | 3216 | 0.72 | 0.90 | **0.64** |
| 1382185_at | **C1qtnf2** | Agonists | 309 | 0.71 | 0.75 | **0.60** |
| 1374307_at | **Camk2n1** | Signaling | 851 | 0.82 | 0.88 | **0.64** |
| 1374275_at | **Car11** | Metabolism | 350 | 0.84 | 0.79 | **0.60** |
| 1371953_at | **Ccng2** | Signaling | 622 | 0.74 | 0.77 | **0.52** |
| 1368976_at | **Cd38** | Receptors | 141 | 0.76 | 1.02 | **0.66** |
| 1373812_at | **Cdkn1b** | Signaling | 2389 | 0.78 | 0.76 | **0.59** |
| 1370225_at | **Cited4** | Transcription | 1104 | 0.87 | 0.74 | **0.58** |
| 1389598_at | **Cln6** | Protein synthesis/modification | 577 | 0.70 | 0.81 | **0.61** |
| 1371629_at | **Cxxc5** | Not established | 2212 | 0.88 | 0.68 | **0.65** |
| 1385630_at | **Ddx26b** | RNA binding/processing | 65 | 1.16 | 1.07 | **0.53** |
| 1383641_at, 1393415_at | **Ednra** | Receptors | 1726, 1230 | 0.93 | 0.82 | **0.65** |
| 1388503_at | **Eid1** | Transcription | 3294 | 0.87 | 0.78 | **0.66** |
| 1391241_at | **Etv1** | Transcription | 164 | 0.82 | 0.87 | **0.65** |
| 1379625_at | **Fam164a** | Not established | 341 | 0.69 | 0.88 | **0.66** |
| 1392510_at | **Fam180a** | Not established | 511 | 0.67 | 0.96 | **0.57** |
| 1374298_at | **Fam40b** | Not established | 239 | 0.64 | 0.79 | **0.46** |
| 1371566_at | **Fbxl22** | Protein synthesis/modification | 4854 | 0.67 | 0.96 | **0.61** |
| 1392886_a_at | **Fcrla** | Receptors | 353 | 0.88 | 0.94 | **0.55** |
| 1376593_at | **Foxo3** | Transcription | 1468 | 0.85 | 0.79 | **0.62** |
| 1385601_at | **Frg1** | RNA binding/processing | 208 | 0.80 | 0.83 | **0.66** |
| 1373838_at | **Fut4** | Protein synthesis/modification | 632 | 1.02 | 0.67 | **0.64** |
| 1388395_at | **G0s2** | Not established | 6041 | 0.68 | 0.83 | **0.47** |
| 1374125_at | **Gata5** | Transcription | 568 | 1.08 | 0.70 | **0.66** |
| 1381469_a_at | **Gigyf1** | Signaling | 394 | 0.82 | 0.81 | **0.63** |
| 1390233_at | **Gli2** | Transcription | 183 | 0.75 | 0.94 | **0.62** |
| 1374541_at | **Gys1** | Metabolism | 1974 | 0.69 | 0.92 | **0.60** |
| 1388608_x_at | **Hba-a1** | Metabolism | 8822 | 0.69 | 0.90 | **0.62** |
| 1371332_at | **Hist1h1c** | DNA regulation | 6296 | 0.76 | 0.79 | **0.57** |
| 1381208_at | **Hist3h2a** | DNA regulation | 89 | 0.61 | 0.68 | **0.50** |
| AFFX_Rat_Hexokinase_3/M_at | **Hk1** | Metabolism | 1149, 1010 | 0.69 | 0.73 | **0.65** |
| 1373992_at | **Iigp1** | Signaling | 435 | 0.72 | 0.83 | **0.64** |
| 1382258_at | **Intron:Arhgef3** | Introns | 176 | 0.97 | 0.92 | **0.65** |
| 1384841_at | **Intron:Ctsc** | Introns | 478 | 0.81 | 0.90 | **0.58** |
| 1396641_at | **Intron:Tmpo** | Introns | 310 | 0.97 | 0.68 | **0.65** |
| 1368486_at | **Irs3** | Signaling | 119 | 0.87 | 0.68 | **0.66** |
| 1384312_at | **Irx1** | Transcription | 177 | 1.19 | 0.82 | **0.64** |
| 1392981_at | **Irx4** | Transcription | 2241 | 1.07 | 0.68 | **0.58** |
| 1369782_a_at | **Kcnj11** | Channels/pumps/transporters | 315 | 0.97 | 0.76 | **0.48** |
| 1368911_at | **Kcnj8** | Channels/pumps/transporters | 986 | 0.78 | 0.74 | **0.54** |
| 1395182_at | **Klhl20** | Cytoskeleton/myofibrillar | 314 | 0.90 | 0.89 | **0.59** |
| 1379462_at | **Ksr1** | Signaling | 386 | 0.78 | 0.86 | **0.65** |
| 1383169_at | **Lifr** | Receptors | 1672 | 0.92 | 0.76 | **0.57** |
| 1376983_at | **LOC682834** | Not established | 64 | 1.03 | 0.67 | **0.58** |
| 1374860_at | **LOC684993** | Not established | 625 | 0.85 | 0.86 | **0.66** |
| 1373172_at | **LOC687356** | Not established | 575 | 0.91 | 0.72 | **0.63** |
| 1373659_at | **LOC688257/LOC689926** | Not established | 445 | 0.98 | 0.76 | **0.60** |
| 1389425_at | **LOC688261** | Not established | 268 | 0.76 | 0.84 | **0.63** |
| 1385299_at | **Lpar4** | Receptors | 367 | 0.88 | 0.75 | **0.62** |
| 1379374_at | **Lppr4** | Signaling | 74 | 0.67 | 0.77 | **0.64** |
| 1389727_at | **Lrrc10** | Cytoskeleton/myofibrillar | 1007 | 0.86 | 0.77 | **0.58** |
| 1385243_at | **Maf** | Transcription | 1395 | 0.75 | 0.76 | **0.62** |
| 1375673_at | **Map3k1** | Signaling | 512 | 0.91 | 0.77 | **0.66** |
| 1370948_a_at | **Marcks** | Cytoskeleton/myofibrillar | 4277 | 0.67 | 0.84 | **0.60** |
| 1388540_at | **Maz** | Transcription | 551 | 0.80 | 0.84 | **0.64** |
| 1393276_at | **Med31** | Transcription | 580 | 0.73 | 0.85 | **0.59** |
| 1371479_at | **Mettl7a** | Metabolism | 620 | 0.75 | 0.79 | **0.57** |
| 1373333_at | **MGC109340** | Protein synthesis/modification | 148 | 0.77 | 0.93 | **0.64** |
| 1367858_at | **Mmp11** | Protein synthesis/modification | 775 | 0.83 | 0.70 | **0.65** |
| 1372597_at | **Mrpl14** | Protein synthesis/modification | 639 | 0.69 | 0.88 | **0.62** |
| 1394182_at | **Mtmr4** | Signaling | 237 | 0.70 | 0.77 | **0.60** |
| 1389474_at | **Mylip** | Protein synthesis/modification | 324 | 0.85 | 0.71 | **0.58** |
| 1390711_at | **Myo9a** | Cytoskeleton/myofibrillar | 169 | 0.82 | 0.93 | **0.61** |
| 1368566_a_at | **Ndufv3** | Metabolism | 3255 | 0.68 | 0.98 | **0.65** |
| 1372959_at | **Nme4** | Metabolism | 385 | 0.73 | 0.83 | **0.57** |
| 1373839_at | **Nope** | Not established | 1095 | 0.75 | 0.89 | **0.65** |
| 1383644_at | **Npr3** | Receptors | 109 | 0.85 | 0.74 | **0.58** |
| 1389554_at | **Nr2f2** | Transcription | 977 | 0.79 | 0.74 | **0.53** |
| 1371849_at | **Nt5dc2** | Not established | 758 | 0.81 | 0.84 | **0.62** |
| 1367507_at | **Nudt22** | Not established | 496 | 0.78 | 0.84 | **0.64** |
| 1390139_a_at | **Obsl1** | Cytoskeleton/myofibrillar | 838 | 0.72 | 0.73 | **0.62** |
| 1377955_at | **Orai2** | Not established | 296 | 0.93 | 0.77 | **0.64** |
| 1391530_a_at | **Oxsm** | Metabolism | 279 | 1.06 | 0.74 | **0.63** |
| 1383327_at, 1383328_x_at | **Pdcd4** | Signaling | 435, 585 | 0.70 | 0.79 | **0.61** |
| 1379211_at | **Pdgfrb** | Receptors | 487 | 1.23 | 0.69 | **0.66** |
| 1382053_at | **Pear1** | Receptors | 825 | 0.89 | 1.02 | **0.66** |
| 1388044_at | **Pfkfb2** | Metabolism | 153 | 0.97 | 0.73 | **0.66** |
| 1368700_at | **Plcl1** | Signaling | 171 | 0.68 | 1.02 | **0.56** |
| 1376494_at | **Pole3** | DNA regulation | 163 | 1.17 | 1.13 | **0.60** |
| 1385393_at, 1393743_at, 1395518_at | **Ppargc1a** | Transcription | 367, 582, 642 | 0.81 | 0.84 | **0.59** |
| 1386653_at, 1390156_a_at | **Prickle2** | Not established | 601, 561 | 0.69 | 0.70 | **0.63** |
| 1376672_at | **Prox1** | Transcription | 681 | 0.90 | 0.82 | **0.62** |
| 1378978_a_at | **Prr12** | Not established | 314, 237 | 0.81 | 0.77 | **0.64** |
| 1370259_a_at | **Pthr1** | Receptors | 637 | 0.84 | 0.82 | **0.65** |
| 1368674_at | **Pygl** | Metabolism | 954 | 0.74 | 0.87 | **0.63** |
| 1377902_a_at | **Rad52** | DNA regulation | 272 | 0.89 | 0.73 | **0.61** |
| 1377187_at | **Rbm12b** | RNA binding/processing | 300 | 0.98 | 0.68 | **0.66** |
| 1382749_at | **Rbm5** | RNA binding/processing | 1648 | 0.88 | 0.69 | **0.64** |
| 1385518_at | **RGD1304931** | Not established | 240 | 0.86 | 1.14 | **0.66** |
| 1392928_at | **RGD1305721** | Not established | 143 | 0.77 | 0.91 | **0.54** |
| 1382235_at | **RGD1306809** | Not established | 327 | 0.77 | 0.89 | **0.66** |
| 1374023_at | **RGD1307170** | Not established | 641 | 0.82 | 0.81 | **0.64** |
| 1372202_at | **RGD1310553** | Not established | 743 | 0.97 | 0.77 | **0.66** |
| 1397579_x_at | **RGD1359127** | Not established | 411 | 0.78 | 0.72 | **0.54** |
| 1388624_at | **RGD1561113** | Not established | 1363 | 0.76 | 0.88 | **0.60** |
| 1373696_at | **RGD1564859** | Not established | 581 | 0.87 | 0.77 | **0.60** |
| 1376927_at | **RGD1564978** | Not established | 304 | 0.89 | 0.74 | **0.60** |
| 1372370_at | **Rpusd4** | RNA binding/processing | 247 | 0.84 | 0.71 | **0.62** |
| 1373427_at | **Rragd** | Signaling | 382 | 0.89 | 0.90 | **0.64** |
| 1385519_at, 1389748_at, 1395294_at | **Runx1t1** | Transcription | 256, 536, 403 | 0.81 | 0.81 | **0.64** |
| 1389437_at | **Sall2** | Transcription | 407 | 0.92 | 0.75 | **0.60** |
| 1372698_at | **Samd1** | Not established | 917 | 0.86 | 0.81 | **0.66** |
| 1367593_at | **Sepw1** | Metabolism | 963 | 0.69 | 0.97 | **0.63** |
| 1390387_at | **Sh3d19** | Not established | 422 | 0.85 | 0.82 | **0.66** |
| 1374932_at | **Similar to Gm6484** | Not established | 272 | 0.63 | 0.64 | **0.40** |
| 1393516_at | **Slc16a12** | Channels/pumps/transporters | 413 | 0.82 | 0.81 | **0.67** |
| 1374200_at | **Slc29a3** | Channels/pumps/transporters | 338 | 0.78 | 0.83 | **0.64** |
| 1383253_at | **Slc30a1** | Channels/pumps/transporters | 1036 | 0.67 | 0.98 | **0.61** |
| 1385171_s_at | **Smtnl2** | Not established | 487 | 0.80 | 0.92 | **0.65** |
| 1379662_a_at, 1386282_x_at | **Snrk** | Signaling | 1659, 307 | 0.76 | 0.88 | **0.55** |
| 1373239_at | **Snx33** | Signaling | 708 | 0.80 | 0.74 | **0.53** |
| 1389460_at | **Socs6** | Signaling | 366 | 0.87 | 0.67 | **0.59** |
| 1390864_at | **Sos1** | Signaling | 288 | 0.87 | 0.81 | **0.63** |
| 1390819_at | **Tef** | Transcription | 614 | 0.87 | 0.96 | **0.64** |
| 1393517_at | **Tmc6** | Not established | 269 | 0.77 | 0.90 | **0.67** |
| 1377857_at | **Tmem196** | Not established | 227 | 0.71 | 1.00 | **0.62** |
| 1384195_at | **Tmem45a** | Not established | 2624 | 0.67 | 0.94 | **0.63** |
| 1373578_at | **Trim2** | Not established | 618 | 0.84 | 0.97 | **0.57** |
| 1378592_at | **Trim59** | Not established | 242 | 0.93 | 0.70 | **0.62** |
| 1378324_at | **Ttc30b** | Not established | 205 | 0.80 | 0.67 | **0.53** |
| 1367953_at | **Tyro3** | Signaling | 318 | 0.79 | 0.80 | **0.65** |
| 1392493_at | **Ubn2** | Not established | 294 | 0.67 | 0.88 | **0.50** |
| 1388919_at | **Unknown** | Unknown | 337 | 0.91 | 0.79 | **0.66** |
| 1377462_at | **Unknown** | Unknown | 197 | 0.89 | 0.86 | **0.66** |
| 1384135_at | **Unknown** | Unknown | 358 | 0.87 | 0.77 | **0.63** |
| 1378602_at | **Unknown** | Unknown | 298 | 0.94 | 0.71 | **0.61** |
| 1389618_at | **Unknown** | Unknown | 957 | 0.88 | 0.86 | **0.60** |
| 1391885_at | **Unknown** | Unknown | 187 | 0.82 | 0.81 | **0.60** |
| 1382228_at | **Unknown** | Unknown | 451 | 0.98 | 0.69 | **0.58** |
| 1398727_at | **Unknown** | Unknown | 1244 | 0.91 | 0.96 | **0.58** |
| 1378808_at | **Unknown** | Unknown | 304 | 0.96 | 0.73 | **0.56** |
| 1386002_at | **Unknown** | Unknown | 179 | 0.84 | 0.69 | **0.53** |
| 1394184_at | **Unknown** | Unknown | 112 | 1.00 | 0.73 | **0.50** |
| 1384269_at | **Unknown** | Unknown | 66 | 0.55 | 0.76 | **0.49** |
| 1387227_at | **Wipf1** | Cytoskeleton/myofibrillar | 1382 | 0.69 | 0.84 | **0.61** |
| 1384029_at | **Xpa** | DNA regulation | 242 | 0.73 | 0.84 | **0.60** |
| 1376376_at | **Zbtb44** | Transcription | 410 | 0.83 | 0.71 | **0.57** |
| 1382199_at | **Zcchc14** | Not established | 762 | 0.88 | 0.68 | **0.67** |
| 1386106_at, 1388672_at | **Zcchc24** | Not established | 622, 1884 | 0.84 | 0.80 | **0.59** |
| 1393120_at | **Zfp251** | Transcription | 465 | 0.93 | 0.79 | **0.65** |
| 1397467_at | **Zfp329** | Transcription | 214 | 0.89 | 0.71 | **0.47** |
| 1393127_at | **Zfp358** | Transcription | 877 | 0.76 | 0.78 | **0.62** |
| 1390148_a_at | **Zfp395** | Transcription | 837 | 0.75 | 0.92 | **0.59** |
| 1393360_at | **Zfp426l** | Transcription | 1095 | 0.92 | 0.72 | **0.62** |
| 1389445_at | **Zfp688** | Not established | 379 | 0.98 | 0.68 | **0.59** |
| 1370589_at | **Zfp709** | Transcription | 131 | 1.02 | 0.64 | **0.55** |
| 1383540_at | **Zfp780b** | Transcription | 256 | 0.97 | 0.85 | **0.64** |
| 1382362_at | **Zfp87** | Transcription | 327 | 0.90 | 0.68 | **0.59** |
| 1372030_at | **Zfyve21** | Not established | 1226 | 0.90 | 0.82 | **0.66** |
| 1390425_at | **Zik1** | Transcription | 169 | 0.98 | 0.79 | **0.61** |
| 1376917_at, 1390945_at | **Znf292** | Transcription | 441, 351 | 0.95 | 0.70 | **0.63** |
| 1384517_at | **Znf496** | Transcription | 871 | 0.73 | 0.73 | **0.63** |
