# Supplementary material for: ERK1/2 Signaling Dominates Over RhoA Signaling in Regulating Early Changes in RNA Expression Induced by Endothelin-1 in Neonatal Rat Cardiomyocytes
Source: PLoS One. 2010 Apr 2;5(4):e10027. doi: 10.1371/journal.pone.0010027 (PMC2848868; doi:10.1371/journal.pone.0010027)
Supplement: Table S7 — Primers used for qPCR validation of microarray data. Nucleotide positions in transcripts are shown in parentheses for each primer. mRNA sequences (accession numbers provided) for established genes were obtained from the Rat Genome Database (http://rgb.mcw.edu, viewed at http://www.ncbi.nlm.nih.gov/entrez). (0.01 MB PDF) [file pone.0010027.s007.pdf]

Table S7. Primers used for qPCR validation of microarray data. Nucleotide positions in transcripts are shown in parentheses for each primer. mRNA sequences (accession numbers provided) for established genes were obtained from the Rat Genome Database (http://rgb.mcw.edu, viewed at http://www.ncbi.nlm.nih.gov/entrez).

Gene	Accession no.	Size (bp)	Forward primer	Reverse primer
Abra	NM_175844	90	CCTTGCACTCCCAGGCAA (691-708)	ACCTGCCTTTCAAGTTGTCCAC (759-780)
Areg	NM_017123	71	TCGCAGCTATTGGCATCATTA (611-633)	TTCTGCTTCTTCATATTCCCTGAA (658-681)
Bmp2	NM_017178	93	CATCACGAAGAAGCCATCG (395-413)	CTCATCAGTAGGGACAGAAC (468-487)
Egr1	NM_012551	117	CTATGAGCACCTGACCACAGAGTC (561-584)	GCAACCGAGTCGTTTGGCT (659-677)
Egr2	NM_053633	121	CTGGAGATGGCATGATCAACA (507-527)	TCTGGTTTCTAGGCGCAGAG (608-627)
Egr3	NM_017086	122	CAATCTGTACCCCGAGGAGATC (66-87)	ACATTCTCTGTAGCCATCTGAGTGTAA (161-187)
Egr4	NM_019137	101	TCCTGGAGGCGACTTCTTGA (317-336)	GCCCTCCAGGAAGCAGGA (399-417)
Ereg	NM_021689	82	CACCGAGAGAAGGATGGAGACT (123-144)	GGGAACCAAGGCAAAGCA (187-204)
Gapdh	NM_002046	113	CCAAGGTCATCCATGACAACTT (552-573)	AGGGGCCATCCACAGTCTT (626-664)
Hbegf	NM_012945	106	GGAGAGTGCAGATACCTGAAGGA (384-406)	GTCAGCCCATGACACCTCTGT (469-489)
IL6	NM_012589	157	GAGTTGTGCAATGGCAATTC (266-285)	ACTCCAGAAGACCAGAGCAG (403-422)
Inhba	NM_017128	94	GAATGAACTCATGGAGCAGACCT (504-529)	AATGCAGTGTCTTCCTGGCTG (577-597)
Klf4	NM_053713	175	TCAAGAGCTCATGCCACCGG (1180-1199)	CTCGCCTGTGTGAGTTCGCA (1335-1354)
Klf6	NM_031642	98	GCTCCCACTTGAAAGCACATC (641-658)	TTCTTGCAAAACGCCACTCA (729-738)
Klf15	NM_053536	74	TGCGGCTGGAGGTTTTCA (1347-1354)	TCACACCCGAGTGAGATCGT (1411-1420)
Slc25a25	NM_145677	80	GACTGCCCATGCTCAATGAGT (2945-2965)	TTTCCTCATCAGGCCATTCTG (3004-3024)
Srf	NM_001109302	81	CATGAAGAAGGCTTATGAGCTGTC (820-843)	TACACATGGCCTGTCTCACTGG (879-900)
